# Linkages between changes in the 3D organization of the genome and transcription during myotube differentiation in vitro

**DOI:** 10.1186/s13395-017-0122-1

**Published:** 2017-04-05

**Authors:** Malina D. Doynova, James F. Markworth, David Cameron-Smith, Mark H. Vickers, Justin M. O’Sullivan

**Affiliations:** 1 Liggins Insitute, University of Auckland, Grafton, Auckland, 1032 New Zealand; 2 Liggins Institute, University of Auckland, Private Bag 92019, Auckland, 1142 New Zealand

**Keywords:** Muscle, Genome organization, Development, Hi-C, Transcriptome

## Abstract

**Background:**

The spatial organization of eukaryotic genomes facilitates and reflects the underlying nuclear processes that are occurring in the cell. As such, the spatial organization of a genome represents a window on the genome biology that enables analysis of the nuclear regulatory processes that contribute to mammalian development.

**Methods:**

In this study, Hi-C and RNA-seq were used to capture the genome organization and transcriptome in mouse muscle progenitor cells (C2C12 myoblasts) before and after differentiation to myotubes, in the presence or absence of the cytidine analogue AraC.

**Results:**

We observed significant local and global developmental changes despite high levels of correlation between the myotubes and myoblast genomes. Notably, the genes that exhibited the greatest variation in transcript levels between the different developmental stages were predominately within the euchromatic compartment. There was significant re-structuring and changes in the expression of replication-dependent histone variants within the HIST1 locus. Finally, treating terminally differentiated myotubes with AraC resulted in additional changes to the transcriptome and 3D genome organization of sets of genes that were all involved in pyroptosis.

**Conclusions:**

Collectively, our results provide evidence for muscle cell-specific responses to developmental and environmental stimuli mediated through a chromatin structure mechanism.

**Electronic supplementary material:**

The online version of this article (doi:10.1186/s13395-017-0122-1) contains supplementary material, which is available to authorized users.

## Background

Skeletal muscle development (myogenesis) is a complex, multistep process that converts multipotent mesodermal cells into myotubes and, subsequently, muscle fibres [1, 2]. This developmental process commences with progenitor proliferation, continues with exit from the cell cycle, early differentiation, alignment and fusion of the mononucleated myoblasts into multinucleated myotubes (late/terminal differentiation) [1–3]. Myogenesis is associated with the stable expression of muscle-specific genes and gene families including the myosin heavy chain (MHC) and the actin gene [4] superfamilies, which contribute to the thick and thin components of the sarcomere in the muscle fibres [5–9], respectively.

Muscle differentiation and cell cycle arrest are tightly regulated and highly interdependent. For example, Myod1 regulates cell cycle arrest by inducing p21 (Cdkn1a), which is a major cyclin-dependent kinase (CDKs) inhibitor; the expression of which results in cell cycle withdrawal [10, 11]. Additional CDK inhibitors (i.e. p21(Cdkn1a), p27(Cdkn1b) [12], p57(Cdkn1c) [11]) and retinoblastoma protein Rb (Rb1) [13–15] are induced during myogenesis to help govern cell cycle withdrawal. The post-mitotic state of differentiated cells is achieved by the expression of CDK inhibitors and, mainly, p21 (Cdkn1a) [10, 11].

At the molecular level, cell fate determination and terminal differentiation of the myogenic lineage-committed cells is managed by a network of muscle-specific helix-loop-helix myogenic regulatory factors (MRFs) [16–19]. These MRFs (e.g. Myod1, Myf5, Myf6 and myogenin) are exclusively expressed in cells committed to the myogenic lineage. In general, MyoD and Myf5 are expressed in proliferating, undifferentiated cells [20, 21]. In contrast, myogenin expression is induced upon early to late muscle differentiation [1, 22–25], while Myf6 is expressed throughout myogenesis [26].

On both the global and local scales, chromatin interactions make an essential contribution to establishing and maintaining the genome organization in the eukaryotic nucleus [27–34]. The formation of particular interactions in response to the presence or absence of cell-type-specific transcription factors (TFs) has been previously reported for a variety of mammalian cells [35–38]. As such, genome organization and nuclear function are interrelated, but the mechanisms that govern the interrelationship are not yet fully elucidated. Despite this, there are several ubiquitous principles for the spatial organization of mammalian genomes [34, 39]. Firstly, chromosomes occupy preferred non-exclusive positions, or territories, in the nuclear space [40, 41]. Secondly, chromosomal sub-regions fold into topologically associating domains (TADs) [34, 39, 42–44] that they are enriched for intra-domain interactions and depleted of inter-domain interactions [34, 39, 42–44]. Finally, contiguous TADs fall into either A or B compartments, which are megabase-sized nuclear domains related to genomic function, that are correlated with early replication and active chromatin (i.e. euchromatin), or late replication and repressed chromatin states (i.e. heterochomatin), respectively [39, 43].

We interrogated the interrelationship between 3D genome organization and gene expression during muscle development in vitro using the mouse C2C12 cell line. C2C12 cells were cultured and harvested as (1) proliferating myoblasts (myoblasts), (2) myotubes, or (3) myotubes that were treated with AraC (a cytidine analogue that is incorporated into newly synthesized DNA [45–49] leading to the termination of DNA elongation, DNA fragmentation [50] and, eventually, cell death) to deplete the culture of undifferentiated myoblasts. The C2C12 cell line is a well-established and extensively studied in vitro model [51–53] derived from serial passage of myoblasts cultured from the thigh muscle of C3H mice after a crush injury [54]. Our results provide evidence for (1) differential and ongoing expression of replicative histone variants within the HIST1 locus during muscle cell development and (2) muscle cell-specific responses to developmental and environmental stimuli mediated through a chromatin structure mechanism.

## Methods

### C2C12 cell culturing

Myoblasts from the skeletal muscle-derived C2C12 cell line were obtained from American Type Culture Collection (ATCC® CRL1772™). All experiments were performed using cells at passage 3.

C2C12 myoblasts were propagated at 37 °C, 5% CO_2_ in Dulbecco’s modified Eagle’s medium (DMEM; high glucose, +pyruvate, +phenol red, +l-glutamine; Gibco® 11995–073) supplemented with 10% foetal bovine serum (FBS; Gibco®) and antibiotics (penicillin 100 U/ml, streptomycin 100 μg/ml) (Gibco®). Myoblasts were plated at a low cellular density of 5 × 10^3^/cm^2^ (to achieve sub-confluent myoblast cultures) or a high cellular density of 2.5 × 10^4^/cm^2^ (to allow cell crowding and myogenic differentiation to occur).

Six replicate T75 culture flasks (Greiner bio-one, 658175, 20 mL media volume) were plated for each experimental condition to achieve cell numbers required for Hi-C. Following 72 h proliferation, cells which were plated at the low density were harvested as sub-confluent myoblast cultures (myoblasts). At the same time (D0), high-density cultures were switched to differentiation media (DMEM, 2% horse serum (HS, Gibco® 16050–122), penicillin 100 U/ml, and streptomycin 100 μg/ml) to induce myotube formation. Following 3 days of differentiation, myotube cultures were either harvested (myotubes) or switched to differentiation media supplemented with 10 μg/mL cytosine β-d-arabinofuranoside (AraC, Sigma, C1768) in order to eliminate undifferentiated myoblasts. AraC media was replaced on day 5 before harvesting the AraC-treated myotubes on day 7 (AraC-treated myotubes). Cell densities at plating and harvesting were as indicated in Additional file 1: Table S1.

### Immunocytochemistry

In 72 h after proliferation, or 3 and 7 days after switch to differentiation media, culture media from the individual wells of the 12-well plates was removed from myoblasts, myotubes and AraC-treated myotubes, respectively, and replaced with fresh pre-warmed DMEM supplemented with 10% FBS or 2% HS, myoblasts and myotubes, respectively, and MitoTracker® Red CMXRos dye. Cells were incubated (37 °C, 30 min) in the presence of MitoTracker® (final conc. 300 nM in 1 mL DMEM), followed by 2 × 5 min incubations in DMEM (1 ml, 37 °C) to remove unbound dye. Cells were fixed in formaldehyde/PBS *w*/*v* (1 ml, final conc. 3.7%, 15 min, 37 °C) and washed with three changes of PBS (1 ml, 5 min, 37 °C each). Fixed cells were permeabilized with Triton X-100/PBS *w*/*v* (1 ml, final conc. 0.1%) for 10 min at RT, washed three times with 1 ml PBS (5 min, RT), blocked in 300 μl of 1% *w*/*v* bovine serum albumin (BSA)/PBS (1 h, RT) and incubated (o/n, 4 °C) in blocking buffer containing primary antibody against sarcomeric myosin (MF20 antibody) (300 μl, 1:20 dilution). In the following day, cells were washed in PBS (5 min, RT) repeated five times and then incubated in Goat anti-Mouse IgG (H + L) Alexa Fluor® 488-conjugated secondary antibody (300 μl diluted 1:200 in PBS) with 300 nM 4′,6-diamidino-2-phenylindole (DAPI; 1 h, RT). Following further washing in PBS (5 min, RT), cells were imaged using the Molecular Devices ImageXpress Micro XLS High-content Screening System equipped with Andor Zyla CMOS camera and ×10/0.3 NA Plan Fluor lens. Images were captured from nine random pre-selected sites in each of three replicate culture wells. Global linear adjustments to image fluorescent signal brightness/contrast were made in ImageJ.

### Image capture analysis

High-content screening (HCS) of C2C12 myoblasts was performed throughout the time course of differentiation using a Molecular Devices ImageXpress Micro XLS automated wide-field microscope.

MetaXpress software (version 5.3.0.5, Molecular Devices) was used for automated image analysis of the extent of myogenic differentiation. Briefly, the Multi-Wavelength Cell Scoring analysis journal was used to quantify the differentiation index (% differentiation) using automated counts of the total number of DAPI-stained nuclei per field (DAPI; wavelength (W) 1) and the percentage of W1 counts located within a sarcomeric myosin (MF20)-positive cell body (Alexa Fluor 488; W2). Global linear adjustments to image fluorescent signal brightness/contrast were made to all pixels within an image in ImageJ software in representative images.

### Preparation of C2C12 Hi-C libraries

Hi-C libraries were prepared as described previously [43], with minor modifications (Additional file 2). For the preparation of the Hi-C libraries, cells were grown in T-75 flasks. Two biological replicates of the C2C12 myoblasts, myotubes and AraC-treated myotubes were prepared from C2C12 cells obtained from different source vials seeded on different days.

### Hi-C data analysis

#### Mapping of the Hi-C libraries and generation of QC reports

We used HiCUP (hicup_v0.5.3) (http://www.bioinformatics.babraham.ac.uk/projects/hicup/) pipeline to analyse the Hi-C libraries. The pipeline was fed with the forward and the reverse reads generated from the sequencing for each of the six libraries. Sequencing reads were mapped to the reference genome (Mus_musculus_GRCh38) using bowtie aligner to generate BAM files. BAM files obtained this way have the corresponding forward and reverse reads of a sequenced DNA fragment mapped to the reference genome as a pair (di-tag). The choice to use HiCUP software for Hi-C data mapping was motivated by the ability of the pipeline to execute a variety of filtering steps (e. g. removal of contiguous sequences, wrong size, re-ligation, same fragment-internal, same fragment-dangling ends, same fragment-circularized). Additionally, the HiCUP pipeline provides summary statistics for each stage of data processing, enabling precise identification of potential problems regarding the quality of the Hi-C libraries.

#### Hi-C analysis

We employed the HOMER Hi-C software pipeline (http://homer.ucsd.edu/homer/interactions/index.html) [55] and HiCUP pipelines to generate interaction matrices, to perform identification of A and B compartments and to determine significant interactions (Additional file 2).

Venn diagrams were plotted using R (‘Vennerable’ package) [56].

#### RNA extraction

C2C12 cells were differentiated in 12 W Multiwell Plates (Greiner bio-one, 665180, 1 mL media/well) for RNA extraction. RNA was extracted using Trizol (Invitrogen) and RNAeasy Mini Kit (Qiagen) (Additional file 2). RNA purity was evaluated by NanoDrop (ND-1000 spectrophotometer; 260/280 and 260/230 ratios). Equal RNA amounts extracted from three separate wells were combined (5 μg total) to form a representative RNA sample. RNA integrity was determined using an Agilent RNA 6000 Nano Kit on an Agilent 2100 Bioanalyzer Instrument. The RNA integrity number (RIN) was consistent with high-quality RNA and ranged between 9.4 and 10. RNA samples (500 ng) were run on an agarose gel (1% (*w*/*v*)) to confirm the absence of DNA. Paired-end sequencing reads were generated by sequencing on an Illumina (HiSeq 2500) platform (BGI).

#### RNA-seq data analyses

Sequenced RNA reads had Phred quality scores ≥ 24 and were not trimmed (http://www.bioinformatics.babraham.ac.uk/projects/fastqc/). Genes that were differentially expressed were identified using TopHat (TopHat v2.0.9) (http://ccb.jhu.edu/software/tophat/index.shtml) and Cufflinks (cufflinks v2.1.1) [57]. Paired RNA reads were aligned to a reference genome (UCSC-mm10.fa) and the splice sites of the genes identified by providing a reference transcriptome (UCSC-mm10.gtf file) in TopHat (with parameters -*r* 200 -*p* 32). Significantly differentially expressed genes were identified using the Cuffdiff function of the Cufflinks package. Transcript levels were plotted as log10 of the fragments per kilobase of transcript per million mapped reads (FPKM) values +1 using ‘cummerbund’ package from R [56].

#### GO analyses

Genes that fell within the top or bottom 10% of the significantly differentially expressed transcript levels were subjected to term enrichment analysis for ‘biological process’ using GOTermFinder (http://go.princeton.edu/cgi-bin/GOTermFinder [58]. The *p* value cut-off was set at 0.05 and gene lists queried against the Mouse Genome Informatics (MGI) database.

#### KEGG pathway analyses

Differentially expressed genes were queried against Kyoto Encyclopedia of Genes and Genomes (KEGG) pathways using DAVID (the database for annotation, visualization and integrated discovery) [59] with default parameters.

## Results

### Morphological characterization of differentiated muscle cells

**Fig. 1:**
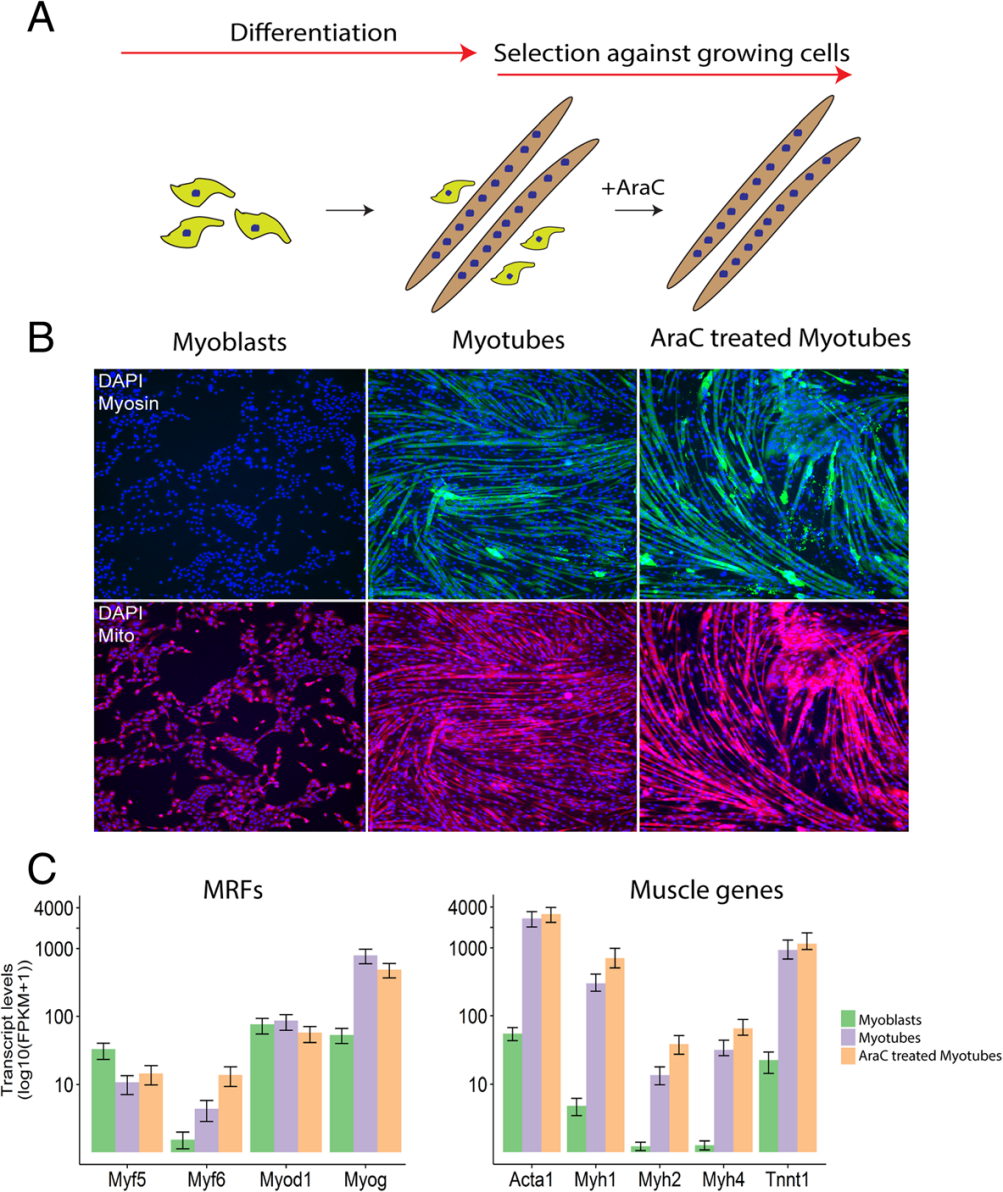
Myoblasts were successfully differentiated into myotubes. **a** Muscle progenitor cells (myoblasts) were differentiated into myotubes in presence of differentiation media. This cell population was predominately comprised of myotubes although there were some myoblasts present. AraC treatment of the myotubes was undertaken to remove undifferentiated myoblasts. **b** Double immunostained images of cells harvested at different stages of myogenic progression. The AraC treatment resulted in the apparent removal of the myoblasts from the terminally differentiated myotubes as evidenced by the presence of cell-free patches in the AraC-treated cultures. Cells were immunostained for myosin (*green*), nuclei (*blue*) and mitochondria (*red*). **c** *Myog* and other skeletal muscle-specific genes (*Acta1*, *Myh1*, *Myh2*, *Myh4*, *Tnnt1*) are markedly upregulated in the myotubes with and without AraC treatment when compared to myoblasts. This indicates successful differentiation at the level of muscle-specific molecular markers. Transcript levels are shown as the mean of the log FPKM for the biological repeats ± SE

C2C12 cells were differentiated into myotubes (Fig. 1a) as evidenced by (1) the presence of sarcomeric myosin and (2) mitochondrial networks on days 3 and 7 following the medium switch (Fig. 1b). The percentage of differentiation was calculated by counting the number of DAPI-stained nuclei located within the myosin positive cell body, from nine randomly pre-selected fields of view (Additional file 3: Figure S1). The total number of DAPI staining nuclei counted within each field of view decreased from that observed at confluence (i.e. 2704). Following the addition of AraC, the total nuclei per field (i.e. 1303; Additional file 3: Figure S1A) decreased further with the loss of residual myoblasts. In contrast, the percentage of nuclei located within the myosin positive cell body (i.e. the percentage of differentiation) showed a statistically significant increase (*p* < 0.05, one-way ANOVA) to 76.2% over the 7-day culture period (Additional file 3: Figure S1). Notably, there was no significant difference in the percentage of differentiation between day 5 (81.7%), where myotubes reached their maximum percentage of differentiation, and day 7 (76.2%) cultures. Thus, we concluded that there was little morphological difference between myotubes on day 5 or day 7 differentiation.

### Global transcriptome changes were consistent with muscle differentiation

Total RNA was extracted from myoblasts, myotubes and AraC-treated myotubes and sequenced to determine differences in the whole transcriptome expression profiles. The RNA integrity numbers (RIN) for the RNA samples ranged between 9.45 and 10 (Additional file 1: Table S2). High-throughput sequencing data from myoblasts, myotubes and AraC-treated myotubes generated between 7,669,926 and 7,972,981 100 bp paired reads for the individual replicates (Additional file 1: Table S2). Ninety-two percent of the reads mapped to the mouse reference genome, consistent with the sequenced RNA libraries being of high quality. Moreover, the high correlation in FPKM values between the biological replicates (*r* = 0.98 and 0.99, Additional file 3: Figure S2) for all genes tested was consistent with high reproducibility.

Differentially expressed genes were determined using Cuffdiff (Additional file 4 and Additional file 6). There was significant upregulation of muscle regulatory factors including myogenin (i.e. *Myog*) and muscle-specific genes (i.e. *Acta1*, *Myh1*, *Myh2*, *Myh4* and *Tnnt1*) in the myotubes relative to myoblasts (Fig. 1c; Additional file 4 and Additional file 6). The observed increase in expression for all muscle-specific genes that were tested continued following AraC treatment of the myotubes. These observations were consistent with those of Trapnell et al. who differentiated C2C12 myoblasts under similar conditions to those used here (Additional file 5 and Additional file 6) [57]. Collectively, our morphological and transcriptional analyses were consistent with the successful differentiation of C2C12 myoblasts into myotubes.

A Gene Ontology (GO) analysis revealed significant enrichment for terms related to muscle function and muscle development in the top 10% of significantly upregulated genes during myotube development (Additional file 1: Table S4 and Additional file 7). Similar GO enrichment was observed in the AraC-treated myotubes (Additional file 1: Table S5 and Additional file 8). In contrast, a GO analysis on the 10% most downregulated genes following myotube development or AraC treatment of myotubes identified enrichment for GO terms related to cell cycle and cell cycle regulation (Additional file 1: Table S4 and S5).

AraC treatment of myotubes correlated with increases in transcript numbers for genes that were related to platelet-derived growth factor, response to stimulus and response to cytokines (Additional file 1: Table S6). Moreover, KEGG pathway screening [60] revealed significant enrichment for genes in the cytosolic DNA-sensing pathway (*p* = 0.049) due to the presence of the *Zbp1*, *Csp1* and *Irf7* genes within the upregulated gene set (Additional file 3: Figure S3). The set of the 10% most significantly downregulated genes following AraC treatment of myotubes was enriched for GO terms related to developmental process (Additional file 1: Table S6). These results are consistent with the supposed mode of action of AraC, specifically its incorporation into newly synthesized DNA [45–49] leading to the termination of DNA elongation, DNA fragmentation [50] and, eventually, cell death in growing cells.

### Global genome structure was similar between myoblasts and myotubes

The 3D structure of the genome reflects the underlying nuclear processes, including transcription, that are active in the cell. Therefore, we used the ‘diluted’ Hi-C method [43] to capture the 3D organization of the genome in myoblasts, myotubes and AraC-treated myotubes, in two biological replicates. Between 188 × 10^6^ and 292 × 10^6^, 150 bp paired-end reads were sequenced per library. Sequenced reads were mapped to the mouse reference genome (mm10) using HiCUP [61].

The quality of the Hi-C libraries was checked in HiCUP. Statistics on (1) read alignment, (2) percentage of artefacts and (3) distribution of distances between individual tags (Additional file 1: Table S7) were consistent with high-quality Hi-C libraries (Additional file 3: Figure S4–S9). There was a high number of duplicated di-tags identified during library processing. High numbers of duplicated di-tags do not affect the quality of the Hi-C libraries; however, deduplication resulted in a decrease in the total number of unique di-tags to 19–45 million for the different replicates and limits the resolution of the subsequent analyses to 400 kb. Thus, analyses of changes in the 3D organization of the genome were limited to 400 kb genomic blocks and could not be attributed to individual mouse genes, which are ~62 kb in size on average.

Interaction matrices were generated for each replicate at 500 kb resolution in HOMER [55]. Interaction matrices for biological replicates were highly correlated: myoblasts, *r* = 0.8; myotubes, *r* = 0.93; and AraC-treated myotubes, *r* = 0.90 (Additional file 1: Table S8). Given the high reproducibility between biological replicates, Hi-C libraries for the biological replicates were pooled. The total number of valid di-tags in the pooled libraries were as follows: myoblasts–36,390,904; myotubes–46,407,229; AraC-treated myotubes–29,634,069. Interaction matrices (400 kb resolution) were generated from the pooled biological replicates. The interaction matrices were highly correlated (i.e. myoblasts–myotubes, *r* = 0.83; myoblasts–AraC-treated myotubes, *r* = 0.78; myotubes–AraC-treated myotubes, *r* = 0.93; Pearson correlation). All analyses were performed on the pooled matrices unless otherwise stated.

### There were extensive A/B compartment switches for specific genomic regions

The identification of A and B compartments is a standard procedure for the analysis of Hi-C data and represents the application of a principal component analyses of the interaction matrices [34, 38, 42, 43, 62, 63]. A and B compartments have been previously shown to correspond relatively well to active and inactive genomic regions [43, 64]. Genomic regions which have positive values for the first principal component (PC1) represent the A (active) compartment, and genomic regions which have negative PC1 values represent the B (inactive) compartment [43].

**Fig. 2:**
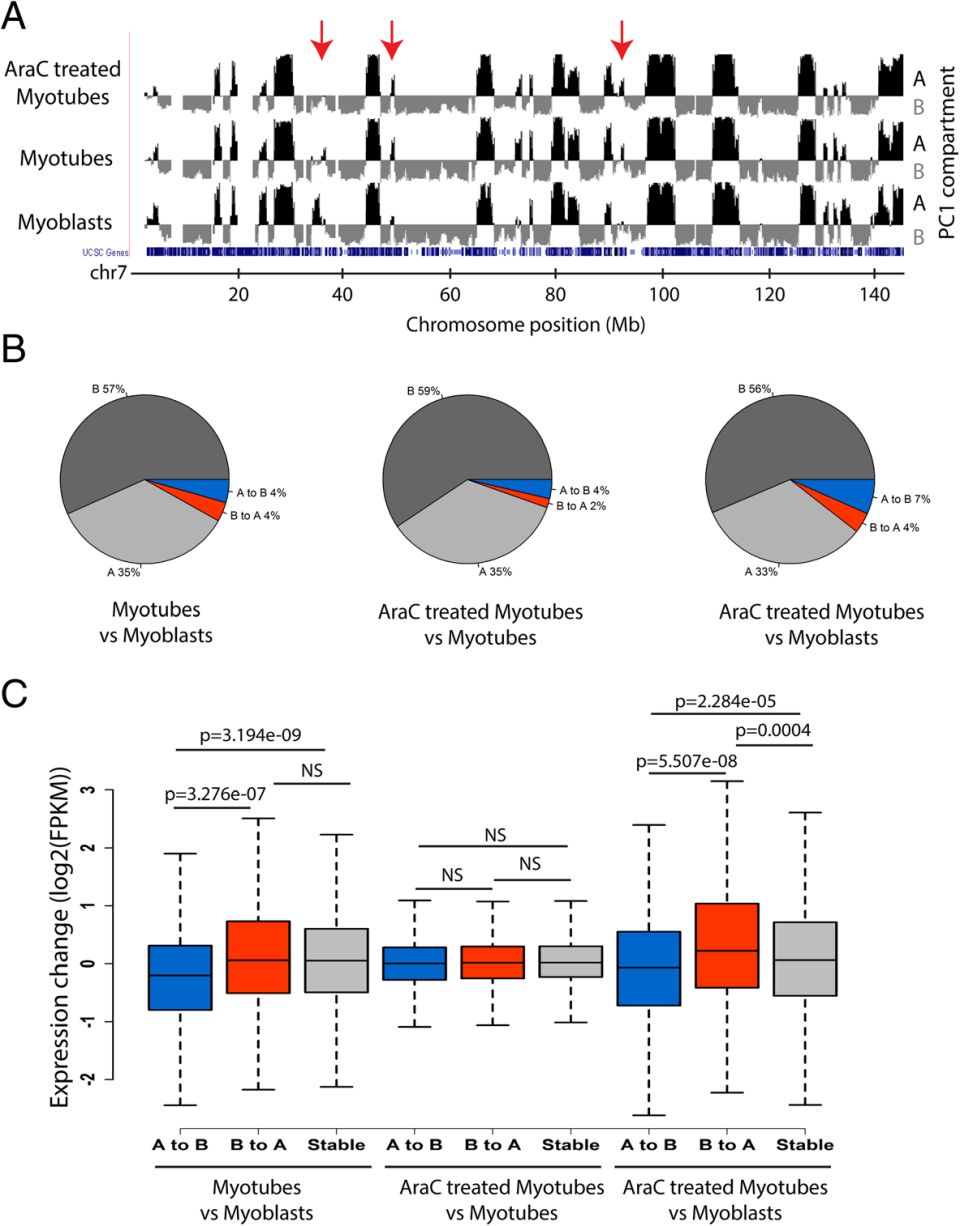
Switches between the A and B compartments were observed in pairwise comparisons between the cell populations. **a** The PC1 values defined using the Hi-C correlation matrices of the three conditions for chr 7 are plotted on the UCSC genome browser. Examples of local changes in the PC1 values between the three conditions are marked with red arrows. **b** Myogenesis is accompanied by significant changes in chromosome topology evident from the fact that 25% of the genome changed compartment status in at least one of the conditions. Comparisons are done only for bins which have detectable PC1 values in the three conditions (6284 bins in total). **c** Genes which changed from residing in A compartment to B compartment reduced their average expression levels, whereas genes which changed from residing in B compartment to A compartment increased their average expression levels for myotube-myoblast and AraC-treated myotube-myoblast comparisons. (*p* values by Wilcoxon test, outliers not plotted). Genes which have expression change of 0 are excluded from the analyses

We evaluated the degree of plasticity of the A and B compartments during myotube development and found that 8% of the genome changed compartment residence (Fig. 2a, b; Additional file 9). Switching from an A to B compartment during differentiation was significantly correlated (*p* < 0.001) with a decrease in gene expression levels (Fig. 2c), consistent with previously published observations in mammalian cells during cell differentiation [38, 42] or de-differentiation [65, 66]. However, while relocating from the B to A compartment was generally associated with an increase in expression, this was only significant in comparisons between the AraC-treated myotubes and myoblasts (Fig. 2c). Notably, changes between A and B compartments did not correlate with changes in expression levels, on the global scale, for comparisons between the AraC-treated myotubes and myotubes (Fig. 2c). Critically, the relative compartment changes between the AraC-treated myotubes and myotubes were similar to those observed when these cells were compared to myoblasts (Fig. 2b). Moreover, 2126 genes were significantly differentially expressed between these cell stages despite their gross phenotypic similarity (Fig. 1). Thus, these results indicate that localization within a particular compartment (i.e. A or B; eu- or heterochromatin) does not automatically dictate expression levels.

**Fig. 3:**
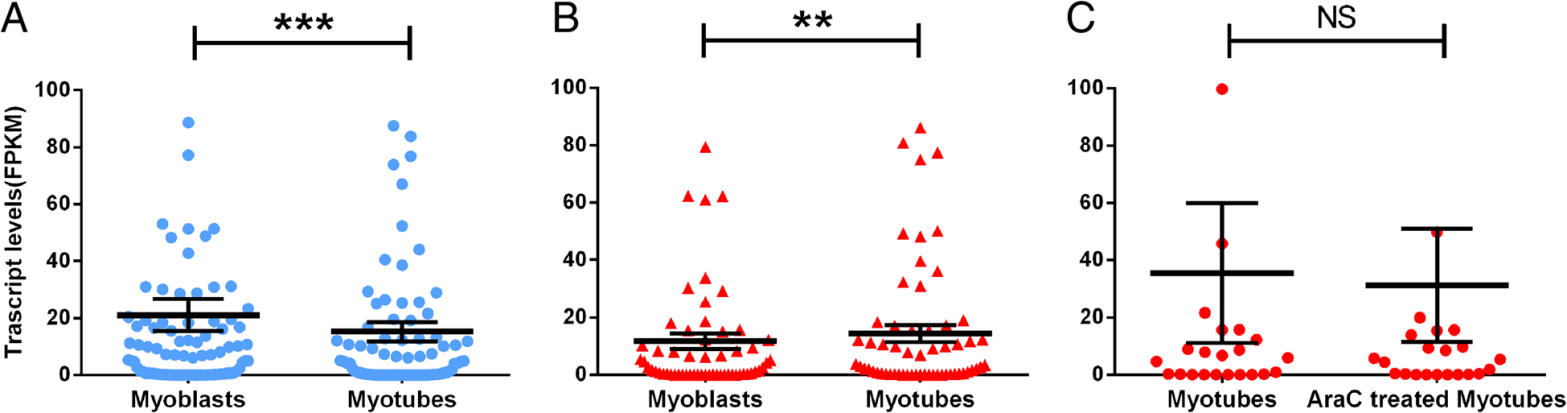
Distribution of transcript levels of genes residing within the confines of the negatively correlated interacting regions in pairwise condition comparisons. Transcript levels (expressed as FPKM) of genes whose **a** TSSs had reduced PC1 values and **b** TSSs showed increases in PC1 values within negatively correlated interacting regions in myotubes vs myoblasts comparison. **c** Transcript levels of genes whose TSSs showed increases in PC1 values within negatively correlated interacting regions in AraC-treated myotubes vs myotubes comparison. Genes which had FPKM values equal to 0 in both conditions are excluded from the analyses; however, genes having FPKM value equal to 0 in one of the conditions and detectable FPKM value in the other condition are included (*p* values–paired Wilcoxon test, *p* < 0.001***, *p* = 0.01**, *NS* not significant). Outliers not plotted

Correlation analyses can be used to identify genomic regions that change their interaction profiles and not just their PC1 values [55]. Central to this analysis is the fact that negatively correlated regions (i.e. *r* < 0) interact with different partners in two conditions. Fifty-five genomic regions (400 kb) were negatively correlated following development of myotubes from myoblasts (Additional file 10). The transcription start sites (TSS) falling within the boundaries of these negatively correlated regions were identified using HOMER and divided into two groups: (1) TSSs whose PC1 values reduced from one condition to the next or (2) TSSs whose PC1 values increased from one condition to the next. The transcript levels of the genes that corresponded to the TSSs in each pool were determined from the transcription data. The differentiation of myoblasts to myotubes resulted in a significant decrease (*p* < 0.001 paired Wilcoxon test) in the transcript levels of 90 genes that were associated with 199 TSSs that had reduced PC1 values (Fig. 3a). In contrast, the 59 genes that were associated with the 112 TSSs that increased their PC1 values (became more open) also significantly increased their transcript levels (i.e. they were upregulated; *p* < 0.01 paired Wilcoxon test; Fig. 3b; Additional file 11). GO analysis identified enrichment for ‘nucleosome assembly’ and ‘chromatin assembly’ (*p* < 0.01; Additional file 1: Table S7) within 11 of the 90 downregulated genes which, with the exception of Cebpg (chr7:35046422–35056573), encoded histone proteins and were located within patch three of the HIST1 cluster on chr 13 (23,600,000–24,000,000 bp). Notably, the Hist1h2bc, Hist1h1c and Histih1a genes within patch three of HIST1 were upregulated upon myotube differentiation (Additional file 4 and Additional file 11). Treatment with AraC resulted in differential expression of specific replicative histone variant genes within the HIST1 cluster. The detection of transcripts that were not previously present (Additional file 1: Table S1) is consistent with the observed changes in expression occurring within the differentiated myotubes and not simply reflecting a change in the population structure (i.e. level of differentiation; Additional file 3: Figure S1). Consistent with the observations by Li et al. [67], the three patches within the HIST1 locus were spatially clustered (Additional file 3: Figure S10). Interestingly, there were only minimal differences in this inter HIST1 clustering between the myotubes and myoblasts indicating it was not responsible for the negative correlation for interactions in this region between the cell types.

The AraC treatment of myotubes resulted in increases to the PC1 values at 41 TSSs. These 41 TSSs overlapped with 24 genes from the transcriptome data (Fig. 3c and Additional file 1: Table S11). Interestingly, there was a non-significant decrease in the overall transcript levels of these 24 genes (Fig. 3c). A GO enrichment of the 24 genes identified an association with pyroptosis (*p* < 0.01; Additional file 1: Table S12). Notably, the genes found in the pyroptosis GO-enriched term are encoded as a multigene family contained within 400 kbp region located on chr13:100,000,000–100,400,000 in the mouse genome.

**Fig. 4:**
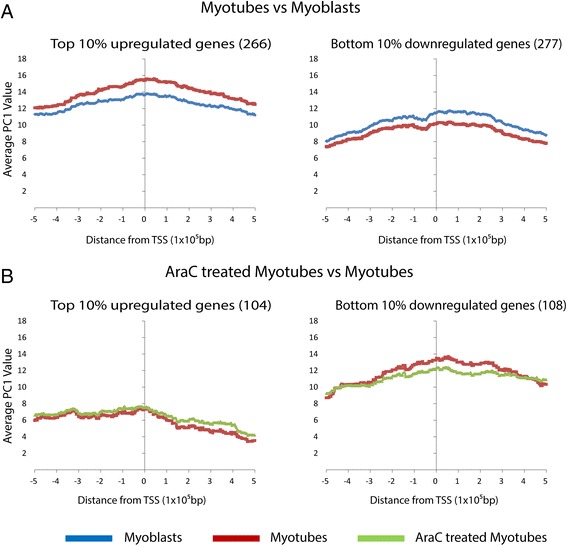
PC1 values across the 10% most upregulated and downregulated genes in comparisons of **a** myotubes vs myoblasts and **b** AraC-treated myotubes vs myotubes. The average PC1 enrichment per 1000 bp bin for the total number of genes in the groups is plotted ±500 kb of the gene TSS. The genomic regions encompassing the upregulated and downregulated genes, for each pairwise comparison, are enriched for positive PC1 values (e.g. reside in A compartment)

The top 10% most upregulated and downregulated transcripts (Additional file 7 and Additional file 8) were underrepresented in the negatively correlated genomic regions. Consistent with this, the genomic regions flanking the 10% most up- and downregulated genes during myogenesis, or treatment with AraC, were enriched for positive PC1 values (Fig. 4). This suggests that the most upregulated and downregulated genes reside within the A compartment during differentiation and AraC treatment of muscle cells.

### Topologically associating domain boundaries

TADs that were conserved between myoblasts, myotubes and AraC-treated myotubes were identified using the Armatus algorithm [68]. Pairwise comparisons show that the numbers of conserved TADs between myotubes and myoblasts were 59 and 57%, respectively (Additional file 3: Figure S11). This result is consistent with earlier findings [38, 39, 66] and the degree of similarity of domain boundaries reported between mouse embryonic stem cells and adult mouse cortex (53.4 and 84%, respectively; [39]); proliferating mouse embryonic stem cells and intermediate neuronal precursor cells (78%; [38]); intermediate neuronal precursor cells and post-mitotic neurons (80%; [38]); and proliferating mouse embryonic stem cells and post-mitotic neurons (74%; [38]); and between human cells [39, 42].

### Gene clustering

**Fig. 5:**
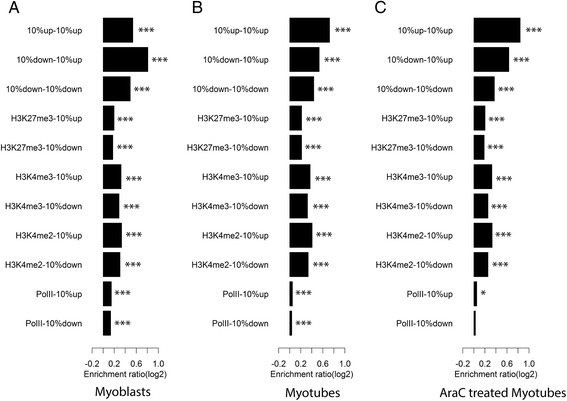
Pairwise feature enrichment at the ends of the significant interactions in **a** myoblasts, **b** myotubes and **c** AraC-treated myotubes. The top 10% and bottom 10% differentially expressed genes from the myotube-myoblast comparison were tested for enrichment in their associations with epigenetic marks for Pol II, H3K4me2, H3K4me3 and H3K27me3 through significant interactions in all three conditions. The enrichment is normalized to the expected number of associations through significant interactions calculated by HOMER, and the corresponding *p* value is calculated by Wilcoxon signed-rank test (*p* < 0.001***, *p* < 0.01**, *p* < 0.05*)

We hypothesized that muscle-specific genes themselves may associate upon muscle differentiation in our in vitro model system for myogenesis. We used HOMER to evaluate clustering of the genes (10%) that had the most upregulated and downregulated transcript levels during myotube differentiation. The top 10% of upregulated genes were significantly clustered in all conditions and the log2 enrichment ratio increased from myoblasts (0.53) to myotubes (0.72; Fig. 5). Consistent with this, there was significant clustering of genes that are involved in muscle cell development (*p* < 0.001), and again, the level of enrichment increased with myotube differentiation (myoblasts, 0.65; myotubes, 0.75; AraC-treated myotubes, 0.76; Additional file 12). Interestingly, the 10% most downregulated genes were also significantly clustered (*p* < 0.001; Fig. 5) in all conditions; however, the log2 enrichment value gradually decreased with development from myoblasts (0.49) to myotubes (0.43) to AraC-treated myotubes (0.37). Again, genes that were annotated as being involved in the ‘mitotic cell cycle’ showed a gradual decrease in clustering with the progression of cell differentiation (enrichment ratio; myoblasts 0.63; myotubes 0.52; and AraC-treated myotubes 0.42, (Additional file 12).

Consistent with our earlier observations that the up- and downregulated genes were present in the A (euchromatic compartment), we observed significant clustering of the top 10% downregulated and upregulated genes in myoblasts (enrichment ratio 0.8; *p* < 0.001). Notably, genes which had no detectable expression in myotubes (10101 genes; Additional file 13) showed low yet significant (*p* < 0.001) non-increasing levels of clustering (enrichment ratio; myoblasts 0.05; myotubes 0.05; Additional file 13) and association with muscle-specific genes (enrichment ratio; myoblasts 0.19; myotubes 0.15; Additional file 13). Collectively, our observations are consistent with genes that are differentially regulated during myotube development being clustered in the progenitor cell line.

### Associations with epigenetic modifications

Remodelling of the epigenetic landscape (e.g. H3K4me2, H3K4me3, H3K27me3) and changes to Pol II loading have been previously shown to accompany myogenesis [53]. We determined the relationship between the chromatin marks, significant chromosomal interactions, and developmentally regulated genes using HOMER and published epigenetic data [53] (Fig. 5; Additional file 14). TADs flanking gene arrays experiencing transcriptional upregulation during differentiation show enrichment for histone marks peculiar for open chromatin and were devoid of histone marks peculiar for compact chromatin (Additional file 3: Figure S12) consistent with previous observations [53, 69]. Overall, the muscle-specific genes showed high levels of clustering with genomic regions that were modified by the epigenetic features that were tested before and after differentiation into myotubes (Fig. 5). Cell cycle-specific genes also clustered with genomic regions harbouring active chromatin histone marks (i.e. H3K4me2, H3K4me3; Fig. 5). This association may be explained by the relatively high retention of these genes within the A (i.e. euchromatic) compartment in myotubes after differentiation and downregulation of these genes (Fig. 4).

There was a decrease in enrichment for interactions that involved associations between the up- and downregulated genes and regions enriched for Pol II upon differentiation to myotubes (Fig. 5). While apparently counterintuitive, the observed decrease in Pol II density at upregulated genes is consistent with (1) a halving in Polr2a transcript levels in myotubes (FPKM = 32.8359) when compared to myoblasts (FPKM = 66.7834) and (2) release of Pol II from pause sites within genes.

## Discussion

We captured the structure of the genome and transcriptome in myoblasts and myotubes using Hi-C and RNA-seq. We observed that the captured genome structures of myoblasts and myotubes were highly correlated, yet there were developmental changes, consistent with previous observations of reduction of the degrees of freedom in genome structure with increasing commitment. Notably, the 10% most upregulated and downregulated differentially expressed genes upon myotube development were enriched for A compartment (i.e. euchromatin) residence and demonstrated significant clustering with other genes that show muscle-specific transcription. Finally, myotubes treated with AraC exhibited changes to the transcript levels and 3D genome organization of sets of genes that were all involved in the same biological process—pyroptosis, a form of inflammatory programmed cell death due to infection.

Histone genes are organized in three multigene complexes (HIST1-3) in the mouse genome with the HIST1 cluster on chromosome 13 being the largest [70]. We observed a developmental switch in genome structure and altered regulation of histone genes within a 400 kb bin on chr13 (23,600,000–24,000,000) that contains the subset of 16 replication-dependent histone genes, including Hist1h1t, that comprise patch three from Hist1 [71]. Consistent with previous observations, the histone gene patches within the HIST1 cluster on chromosome 13 form a multigene cluster that may help coordinate gene expression [67]. The dogma has been that the expression of replication-dependent histones comprising the histone core (H2A, H2B, H3 and H4) is globally downregulated upon cell differentiation as the main ‘consumer’ of histones is newly synthesized DNA [70, 72]. Superficially, such an interpretation would be consistent with our observations as cell differentiation to myotubes corresponds with withdrawal from the cell cycle [73]. However, not all of the histone genes within patch three were downregulated. Specifically, HisIh2bc, Hist1h1a and HistIh1c were upregulated during development of myotubes. The upregulation of both Hist1h1a and HistIh1c may reflect the role of histone H1 in chromatin condensation and the reprogramming of heterochromatic regions we observed. This agrees with recent studies that have shown differential expression of histone variants from within HIST1 and HIST2 during development and in senescent mouse neurons [74]. Higher resolution analyses of the chromatin structural changes that occur within patch three of the HIST1 cluster, using either paused and elongating Pol II-specific ChiA-Pet [75] or circular chromosome conformation capture [76], would help to understand the regulatory cascade that is controlling histone variant expression in differentiated myotubes. The significance of this finding as a mechanism for developmentally significant or post-replication targeting of transcriptional regulation in myotubes remains to be determined. However, the observation that AraC treatment caused additional changes demonstrates that environmental signals can change the patterns of replicative histone expression in differentiated myotubes. The importance of this should not be underestimated given that (1) replication-dependent canonical histone variants undergo specific and rapid exchange during physiological processes (e.g. spermatogenesis (reviewed in [77]); (2) variants in histone H3 have been linked to regulation of gene selection and lineage potential [78]; and (3) mutations in histones have been linked to various developmental disorders and human diseases [77]. Moreover, there are some clear similarities to the control of the developmentally regulated HOX genes (reviewed in [79]). Thus, we propose that investigating these replicative histone variants may help improve our understanding of alterations to gene expression in muscle disorders.

We observed plasticity with respect to the retention of genomic sequences within the A (i.e. euchromatic) and B (i.e. heterochromatic) compartments irrespective of their transcriptional status. However, consistent with earlier observations, not all genes that were present within regions that changed from A to B compartments, or vice versa, significantly changed transcript levels [42, 66]. Thus changing from euchromatic to heterochromatic compartments might not in all cases reflect the immediate gene expression state. Despite this, correlated changes between the nuclear architecture and transcript levels of genes were observed. For example, AraC treatment of myotubes resulted in two forms of changes within a single metabolic pathway (i.e. pyroptosis) which involved coordination of (1) changes to the chromatin compartmentalization and transcript levels (e.g. Naip5, Naip2 and Naip6 that were located in a negatively correlated region on chr13 [100 × 10^6^−100.4 × 10^6^]) and (2) changes to transcript levels for genes (i.e. Zbp1, Csp1 and Irf7) that were independent of changes to compartmentalization remaining in the A (euchromatic) compartment, at the resolution level used here. As such, in this study, we observe a response of the genome structure and transcription to external stimuli, namely, the AraC drug treatment. Numerous studies have shown a link between relatively rapid alterations of the nuclear architecture as a response to external stimuli including (a) thalidomide-dependent alteration of chromatin supra-organization in drug-resistant myeloma cells [80]; (b) marked ribosomal DNA (rDNA) chromatin decondensation and a significant increase in ribosomal gene expression in rye cells subjected to high-temperature stress [81]; and (c) bursts of action potential in cultured hippocampal neurons that cause remodelling of the nucleus to obtain ‘unfoldings’ which are thought to improve the cellular response to calcium signalling [82]. A possible outcome of the overlapping responses in 3D organization and transcription could be increased cell adaptation to the changed environmental cues. With respect to the AraC-dependent induction of the ‘pyroptosis’ genes and activation of the Naip cluster, in case the stimuli persists, could have resulted from the formation of a co-regulated multigene cluster. However, studies of genome conformation are statistical and associational and reveal general tendencies. Thus, it should be remembered that AraC kills dividing cells and, in addition to a direct effect on the structure of the genomes in the treated myotubes, the observed changes could also result from the changes to the population structure (i.e. the ratio of myotubes/myoblasts). While the observed effects are unlikely to be solely due to changes in population structure, additional studies are required to untangle the effects of AraC.

Our observation of reducing TAD conservation with increasing differentiation in mouse cells is consistent with that of Fraser et al. who associated the decreasing trend with a partial reorganization of TADs during differentiation [38]. This in no way contradicts the general observation of a trend for the preservation of TADs between different mammalian cell types [39], during mammalian cell differentiation [38], and during mammalian cell senescence [62, 63]. It is possible that the reason why the levels of conservation we observed were towards the bottom of the published range is due to (1) a biological phenomenon related to the formation of the myotubes which are syncytial cells or (2) the choice of algorithm and absolute conservation of boundary position which we used in our study [68]. Single nuclei approaches [83] could be employed to isolate nucleus-specific effects due to the formation of a syncytium and to confirm the cell-specific remodelling of TADs. This would have important consequences for our understanding of the role of TADs as units structuring the folding of the genome.

The genome and nucleus collectively form a constrained system that is maintained on the boundary of order and chaos [84]. Within this constrained system, genomes are interleaved entities [85] that are spatially organized into hierarchically organized domains of different sizes (e.g. chromosome territories and topologically associated domains) [38, 39]. Moreover, our observations of clustering within the 10% most up- and downregulated genes, which were enriched for residence within the A compartment, are consistent with co-regulation through the formation of multigene clusters [67], some of which (e.g. muscle cell developmental genes) were already present in the myoblasts. The biological reason for this association remains unknown, but it is possible that the association between muscle-specific genes and cell cycle-specific genes acts as a form of ‘crosstalk’ between the cell cycle-specific genes and muscle-specific genes. This is consistent with the known tight coupling between cell proliferation and differentiation [86–88]. Interestingly, it has been recently shown that cell division is a necessary prerequisite for establishing changes in nuclear architecture during myogenesis in human cells [69]. Furthermore, the concomitant expression of these two groups of genes is generally reciprocal [10, 11], and the ‘crosstalk’ in terms of interactions may contribute to such reciprocity. Conversely, muscle-specific genes may occupy a nuclear location close to already recruited transcription machinery (cell cycle-specific genes are highly expressed in myoblasts), which could contribute to increased efficiency of muscle-specific genes transcription once the stimuli for their upregulation is present (e.g. expression of MRFs). This is similar to the active re-location of Myc and Fos genes to pre-assembled transcription factories upon induction of mouse B cells [89].

Chromatin organization and gene control are hierarchical, and epigenetic modifications are widely considered to make a significant contribution to the environment within which genes are regulated. Epigenetic modifications have been shown to cluster in mammalian nuclei [39, 43, 90, 91] and on muscle-specific genes in myoblasts and myotubes [53]. Our results are consistent with this. Interestingly, we noted an enrichment of both active and inactive epigenetic marks with the up- and downregulated genes. This is consistent with observations that these epigenetic modifications act in a combinatorial nature and are not individually indicative of active or inactive chromatin regions [92]. From a biological perspective, it is possible that this spatial co-localization of different epigenetic marks at the muscle cell developmental and cell cycle genes contributes to the relatively easy reversibility [93, 94] and very fine balance within the expression program that separates the differentiation and de-differentiation state in cultured muscle cells. In this scenario, the enrichment of the cell cycle-specific genes in the ‘A’ compartment may therefore reflect the ‘readiness’ of the cell cycle-specific genes to be activated upon the presence of the right transcription cues.

## Conclusions

Our observations are consistent with the precursor C2C12 cell lines being myogenic, i.e. pre-programmed for development into myotubes [54], and having already undergone a degree of genome structural limitation. Moreover, there is extensive evidence for chromatin structure playing a part in the programmed expression of replication-dependent histones following exit from the cell cycle. Finally, this study provides evidence for muscle cell-specific responses to environmental stimuli mediated through a chromatin structure mechanism.

## Additional files

Additional file 1: Table S1. Cell numbers at time of plating and harvesting. **Table S2.** Alignment summary of RNA-seq reads onto the mouse transcriptome (UCSC-mm10.gtf transcriptome file). **Table S3.** The number of significantly differentially expressed genes varied between the conditions (FDR-corrected *p* < 0.05). **Table S4.** The ten top ranked GO term finder results for the 10% of most significantly upregulated and downregulated genes for the myotubes vs myoblasts comparison (*p* values are corrected by Bonferroni correction). **Table S5.** The ten top ranked GO term finder results amongst the top 10% differentially upregulated and downregulated genes between AraC-treated myotubes vs myoblasts (*p* values are corrected by Bonferroni correction). **Table S6.** The ten top ranked GO term finder results amongst the top 10% differentially upregulated and downregulated genes between AraC-treated myotubes vs myotubes (*p* values are corrected by Bonferroni correction). **Table S7.** HiCUP summary report of total number of valid unique di-tags and the distribution of genome distances which separate the individual reads (tags) from the di-tags. **Table S8.** Interaction matrices on 500 kb resolution are highly correlated at the level of biological replicates within and between conditions. **Table S9.** GO terms enriched amongst the genes corresponded to TSS having their PC1 values and transcript levels decreased during the switch from myoblasts to myotubes. **Table S10.** GO terms enriched amongst the genes corresponded to TSS having their PC1 values and transcript levels decreased during the switch from myoblasts to AraC-treated myotubes. **Table S11.** GO terms enriched amongst the genes corresponded to TSS having their PC1 values increased during the switch from myoblasts to AraC-treated myotubes. **Table S12.** GO terms enriched amongst the genes corresponded to TSS having their PC1 values increased during the switch from myotubes to AraC-treated myotubes. (DOCX 43 kb)

Additional file 2: Supplementary methods. (DOCX 53 kb)

Additional file 3: Figure S1. Determination of the total nuclei and percent of differentiation in the course of cell culture. **Figure S2.** Transcript levels, measured as FPKM values, were highly correlated between biological replicates. **Figure S3.** Cytosolic DNA-sensing pathway was enriched (*p* = 0.049) within the gene set containing the top 10% of significantly upregulated genes for AraC-treated myotubes vs myotubes comparison. **Figure S4.** QC report generated after processing of the myoblasts replicate 1 Hi-C library. **Figure S5.** QC report generated after processing of the myoblasts replicate 2 Hi-C library. **Figure S6.** QC report generated after processing of the myotubes (day 3) replicate 1 Hi-C library. **Figure S7.** QC report generated after processing of the myotubes (day 3) replicate 2 Hi-C library. **Figure S8.** QC report generated after processing of the myotubes (day 7 + AraC) replicate 1 Hi-C library. **Figure S9.** QC report generated after processing of the myotubes (day 7 + AraC) replicate 2 Hi-C library. **Figure S10.** Patches of replication-dependent histone variants spatially clustered within the HIST1 locus. **Figure S11.** Distributions of shared and unique interactions and TADs across the three conditions. **Figure S12.** Two consensus TADs visualized on the UCSC genome browser spanning developmentally regulated arrays of muscle genes and their corresponding histone mark signals on chr1. (DOCX 2373 kb)

Additional file 4: FPKM values for all genes. (XLS 6248 kb)

Additional file 5: FPKM for all genes in ﻿Trapnell et al. (XLSX 160 kb)

Additional file 6: FPKM values for ge﻿nes associated with muscle cell differentiation from Figure 1. (XLSX 14 kb)

Additional file 7:﻿ Data for the 10% up or down differentially expressed genes in different conditions. (XLSX 160 kb)

Additional file 8: Gene ontology analysis for muscle development enrichment genes. (XLSX 22 kb)

Additional file 9: Coordinates of regions that switched from A to B compartment. (XLSX 1105 kb)

Additional file 10: Coordinates of negatively correlated regions﻿ in the HiC analysis. (XLSX 19 kb)

Additional file 11: TSS genes Myotubes (Day 3) Myoblasts. (XLSX 83 kb)

Additional file 12: Gene ontology lists for genes by feature enrichment. (XLSX 59 kb)

Additional file 13:﻿ Feature enrichment for undetected genes. (XLSX 436 kb)

Additional file 14: Feature enrichment for top 10% up or down regulated genes. (XLSX 21 kb)

## Abbreviations

AraC: Cytosine β-d-arabinofuranoside
BSA: Bovine serum albumin
CDKs: Cyclin-dependent kinase
D0: Day 0
D5: Day 5
D7: Day 7
DAPI: 4′,6-diamidino-2-phenylindole
DMEM: Dulbecco’s modified Eagle’s medium
FBS: Foetal bovine serum
FPKM: Fragments per kilobase of transcript per million mapped reads
GO: Gene Ontology
HCS: High-content screening
MGI: Mouse Genome Informatics database
MHC: Myosin heavy chain
MRFs: Myogenic regulatory factors
PC1: First principal component
RIN: RNA integrity numbers
TADs: Topologically associating domains
TFs: Transcription factors
TSS: Transcription start sites

## References

[1] Bentzinger CF, Wang YX, Rudnicki MA (2012). Building muscle: molecular regulation of myogenesis. Cold Spring Harb Perspect Biol.

[2] Brand-Saberi B (2015). Vertebrate myogenesis.

[3] Bryson-Richardson RJ, Currie PD (2008). The genetics of vertebrate myogenesis. Nat Rev Genet.

[4] Herman IM (1993). Actin isoforms. Curr Opin Cell Biol.

[5] Barjot C, Cotten M-L, Goblet C, Whalen RG, Bacou F (1995). Expression of myosin heavy chain and of myogenic regulatory factor genes in fast or slow rabbit muscle satellite cell cultures. J Muscle Res Cell Motil.

[6] Weiss A, McDonough D, Wertman B, et al. Organization of human and mouse skeletal myosin heavy chain gene clusters is highly conserved. Proc Natl Acad Sci U. S. A. 1999;96(6):2958–63. 10.1073/pnas.96.6.2958 PMC15877 10077619

[7] Miller JB (1990). Expression of myosin heavy chain isoforms, MyoD1, and myogenin. J Cell Biol.

[8] Schiaffino S, Reggiani C (1994). Myosin isoforms in mammalian skeletal muscle. J Appl Physiol.

[9] Weiss A, Leinwand L (1996). The mammalian myosin heavy chain gene family. Annu Rev Cell Dev Biol.

[10] Waga S, Hannon GJ, Beach D, Stillman B (1994). The p21 inhibitor of cyclin-dependent kinases controls DNA replication by interaction with PCNA. Nature.

[11] Zhang P, Wong C, Liu D, Finegold M, Harper JW, Elledge SJ (1999). p21CIP1 and p57KIP2 control muscle differentiation at the myogenin step. Genes Dev.

[12] Møller MB (2000). P27 in cell cycle control and cancer. Leuk Lymphoma.

[13] De Falco G, Comes F, Simone C (2006). pRb: master of differentiation. Coupling irreversible cell cycle withdrawal with induction of muscle-specific transcription. Oncogene.

[14] Wang J, Guo K, Wills KN, Walsh K (1997). Rb functions to inhibit apoptosis during myocyte differentiation. Cancer Res.

[15] Weinberg RA (1999). The retinoblastoma protein and cell cycle control. Cell.

[16] Braun T, Buschhausen-Denker G, Bober E, Tannich E, Arnold HH (1989). A novel human muscle factor related to but distinct from MyoD1 induces myogenic conversion in 10T1/2 fibroblasts. EMBO J.

[17] Lassar AB, Skapek SX, Bennett N (1994). Regulatory mechanisms that coordinate skeletal muscle differentiation and cell cycle withdrawl. Curr. Opin. Cell Biol..

[18] Sabourin LA, Rudnicki MA (2001). The molecular regulation of myogenesis. Clin Genet.

[19] Weintraub H, Davis R, Tapscott S, Thayer M, Krause M, Benezra R, Blackwell T, Turner D, Rupp R, Hollenberg S (1991). The myoD gene family: nodal point during specification of the muscle cell lineage. Science (80-).

[20] Smith TH, Block NE, Rhodes SJ, Konieczny SF, Miller JB (1993). A unique pattern of expression of the four muscle regulatory factor proteins distinguishes somitic from embryonic, fetal and newborn mouse myogenic cells. Development.

[21] Weintraub H (1993). The MyoD family and myogenesis: redundancy, networks, and thresholds. Cell.

[22] Rawls A, Morris JH, Rudnicki M, Braun T, Arnold HH, Klein WH, Olson EN (1995). Myogenin’s functions do not overlap with those of MyoD or Myf-5 during mouse embryogenesis. Dev Biol.

[23] Cao Y, Kumar RM, Penn BH, Berkes C, Kooperberg C, Boyer L, Young R, Tapscott SJ (2006). Global and gene-specific analyses show distinct roles for Myod and Myog at a common set of promoters. EMBO J.

[24] Wang Y, Jaenisch R (1997). Myogenin can substitute for Myf5 in promoting myogenesis but less efficiently. Development.

[25] Rajan S, Dang HCP, Djambazian H, Zuzan H, Fedyshyn Y, Ketela T, Moffat J, Hudson TJ, Sladek R (2012). Analysis of early C2C12 myogenesis identifies stably and differentially expressed transcriptional regulators whose knock-down inhibits myoblast differentiation. Physiol Genomics.

[26] Hinits Y, Osborn DPS, Carvajal JJ, Rigby PWJ, Hughes SM (2007). Mrf4 (myf6) is dynamically expressed in differentiated zebrafish skeletal muscle. Gene Expr Patterns.

[27] Rodley CDM, Bertels F, Jones B, O’Sullivan JM (2009). Global identification of yeast chromosome interactions using genome conformation capture. Fungal Genet Biol.

[28] Grand RS, Pichugina T, Gehlen LR, Jones MB, Tsai P, Allison JR, Martienssen R, O’Sullivan JM (2014). Chromosome conformation maps in fission yeast reveal cell cycle dependent sub nuclear structure. Nucleic Acids Res.

[29] Pichugina T, Sugawara T, Kaykov A, Schierding W, Masuda K, Uewaki J, Grand RS, Allison JR, Martienssen RA, Nurse P (2016). A diffusion model for the coordination of DNA replication in Schizosaccharomyces pombe. Sci Rep.

[30] Gheldof N, Smith EM, Tabuchi TM, Koch CM, Dunham I, Stamatoyannopoulos JA, Dekker J (2010). Cell-type-specific long-range looping interactions identify distant regulatory elements of the CFTR gene. Nucleic Acids Res.

[31] Naumova N, Smith EM, Zhan Y, Dekker J (2012). Analysis of long-range chromatin interactions using chromosome conformation capture. Methods.

[32] Smallwood A, Ren B (2013). Genome organization and long-range regulation of gene expression by enhancers. Curr Opin Cell Biol.

[33] De S, Michor F (2011). DNA replication timing and long-range DNA interactions predict mutational landscapes of cancer genomes. Nat Biotechnol.

[34] Rao SSP, Huntley MH, Durand NC, Stamenova EK, Bochkov ID, Robinson JT, Sanborn AL, Machol I, Omer AD, Lander ES (2014). A 3D Map of the human genome at kilobase resolution reveals principles of chromatin looping. Cell.

[35] Jing H, Vakoc C, Ying L, Mandat S, Wang H (2008). Exchange of GATA factors mediates transitions in looped chromatin organization at a developmentally regulated gene locus. Mol Cell.

[36] Song SH, Hou C, Dean A (2007). A positive role for NLI/Ldb1 in long-range beta-globin locus control region function. Mol Cell.

[37] De Belle I, Cai S, Kohwi-Shigematsu T (1998). The genomic sequences bound to special AT-rich sequence-binding protein 1 (SATB1) in vivo in Jurkat T cells are tightly associated with the nuclear matrix at the bases of the chromatin loops. J Cell Biol.

[38] Fraser J, Ferrai C, Chiariello AM, Schueler M, Rito T, Laudanno G, Barbieri M, Moore BL, Kraemer DC, Aitken S (2015). Hierarchical folding and reorganization of chromosomes are linked to transcriptional changes in cellular differentiation. Mol Syst Biol.

[39] Dixon JR, Selvaraj S, Yue F, Kim A, Li Y, Shen Y, Hu M, Liu JS, Ren B (2012). Topological domains in mammalian genomes identified by analysis of chromatin interactions. Nature.

[40] Cremer T, Cremer M (2010). Chromosome territories. Cold Spring Harb Perspect Biol.

[41] Cremer T, Cremer M, Dietzel S, Müller S, Solovei I, Fakan S (2006). Chromosome territories—a functional nuclear landscape. Curr Opin Cell Biol.

[42] Dixon JR, Jung I, Selvaraj S, Shen Y, Antosiewicz-Bourget JE, Lee AY, Ye Z, Kim A, Rajagopal N, Xie W (2015). Chromatin architecture reorganization during stem cell differentiation. Nature.

[43] Lieberman-Aiden E, van Berkum NL, Williams L, Imakaev M, Ragoczy T, Telling A, Amit I, Lajoie BR, Sabo PJ, Dorschner MO (2009). Comprehensive mapping of long-range interactions reveals folding principles of the human genome. Science.

[44] Sexton T, Yaffe E, Kenigsberg E, Bantignies F, Leblanc B, Hoichman M, Parrinello H, Tanay A, Cavalli G (2012). Three-dimensional folding and functional organization principles of the Drosophila genome. Cell.

[45] Gray GD, Nichol FR, Mickelson MM, Camiener GW, Gish DT, Kelly RC, Wechter WJ, Moxley TE, Neil GL (1972). Immunosuppressive, antiviral and antitumor activities of cytarabine derivatives. Biochem Pharmacol.

[46] Cohen SS (1977). The mechanisms of lethal action of arabinosyl cytosine (araC) and arabinosyl adenine (araA). Cancer.

[47] Pallavicini MG (1984). Cytosine arabinoside: molecular, pharmacokinetic and cytokinetic considerations. Pharmacol Ther.

[48] Prakasha Gowda AS, Polizzi JM, Eckert KA, Spratt TE (2010). Incorporation of gemcitabine and cytarabine into DNA by DNA polymerase beta and ligase III/XRCC1. Biochemistry.

[49] Garcia-Diaz M, Murray MS, Kunkel T, Chou K-M (2010). Interaction between DNA polymerase lambda and anticancer nucleoside analogs. J Biol Chem.

[50] Mikita T, Beardsley GP (1988). Functional consequences of the arabinosylcytosine structural lesion in DNA. Biochemistry.

[51] Bischoff R (1986). Proliferation of muscle satellite cells on intact myofibers in culture. Dev Biol.

[52] Blais A, Tsikitis M, Acosta-Alvear D, Sharan R, Kluger Y, Dynlacht BD (2005). An initial blueprint for myogenic differentiation. Genes Dev.

[53] Asp P, Blum R, Vethantham V, Parisi F, Micsinai M, Cheng J, Bowman C, Kluger Y, Dynlacht BD (2011). Genome-wide remodeling of the epigenetic landscape during myogenic differentiation. Proc Natl Acad Sci.

[54] Yaffe D, Saxel O (1977). Serial passaging and differentiation of myogenic cells isolated from dystrophic mouse muscle. Nature.

[55] Heinz S, Benner C, Spann N, Bertolino E, Lin YC, Laslo P, Cheng JX, Murre C, Singh H, Christopher K (2011). Simple combinations of lineage-determining transcription factors prime cis-regulatory elements required for macrophage and B cell identities. Mol Cell.

[56] R Core Team (2013). R: a language and environment for statistical computing.

[57] Trapnell C, Williams B, Pertea G, Mortazavi A, Kwan G, van Baren MJ, Salzberg SL, Wold BJ, Pachter L (2010). Transcript assembly and quantification by RNA-Seq reveals unannotated transcripts and isoform switching during cell differentiation. Nat Biotechnol.

[58] Boyle EI, Weng S, Gollub J, Jin H, Botstein D, Cherry JM, Sherlock G (2004). GO::TermFinder—open source software for accessing Gene Ontology information and finding significantly enriched Gene Ontology terms associated with a list of genes. Bioinformatics.

[59] Huang DW, Lempicki RA, Sherman BT (2009). Systematic and integrative analysis of large gene lists using DAVID bioinformatics resources. Nat Protoc.

[60] Kanehisa M, Goto S (2000). KEGG: Kyoto encyclopedia of genes and genomes. Nucleic Acids Res.

[61] Wingett SW, Schoenfelder S, Furlan-Magaril M, Nagano T, Fraser P, Andrews S (2015). HiCUP: pipeline for mapping and processing Hi-C data. F1000Research.

[62] Criscione S, De Cecco M, Siranosian B (2016). Reorganization of chromosome architecture in replicative cellular senescence. Biomolecules.

[63] Chandra T, Ewels PA, Schoenfelder S, Furlan-Magaril M, Wingett SW, Kirschner K, Thuret JY, Andrews S, Fraser P, Reik W (2015). Global reorganization of the nuclear landscape in senescent cells. Cell Rep.

[64] Ryba T, Hiratani I, Lu J, Itoh M, Kulik M, Zhang J, Schulz TC, Robins AJ, Dalton S, Gilbert DM (2010). Evolutionarily conserved replication timing profiles predict long-range chromatin interactions and distinguish closely related cell types. Genome Res.

[65] Beagan JA, Gilgenast TG, Kim J, Plona Z, Norton HK, Hu G, Hsu SC, Shields EJ, Lyu X, Apostolou E (2016). Local genome topology can exhibit an incompletely rewired 3D-folding state during somatic cell reprogramming. Cell Stem Cell.

[66] Krijger PHL, Di Stefano B, de Wit E, Limone F, van Oevelen C, de Laat W, Graf T (2016). Cell-of-origin-specific 3D genome structure acquired during somatic cell reprogramming. Cell Stem Cell.

[67] Li G, Ruan X, Auerbach RK, Sandhu KS, Zheng M, Wang P, Poh HM, Goh Y, Lim J, Zhang J (2012). Extensive promoter-centered chromatin interactions provide a topological basis for transcription regulation. Cell.

[68] Filippova D, Patro R, Duggal G, Kingsford C (2014). Identification of alternative topological domains in chromatin. Algorithms Mol Biol.

[69] Neems DS, Garza-Gongora AG, Smith ED, Kosak ST (2016). Topologically associated domains enriched for lineage-specific genes reveal expression-dependent nuclear topologies during myogenesis. Proc Natl Acad Sci.

[70] Marzluff WF, Gongidi P, Woods KR, Jin J, Maltais LJ (2002). The human and mouse replication-dependent histone genes. Genomics.

[71] Wang ZF, Krasikov T, Frey MR, Wang J, Matera AG, Marzluff WF (1996). Characterization of the mouse histone gene-cluster on chromosome-13–45 histone genes in 3 patches spread over 1 mb. Genome Res.

[72] Ewen ME (2000). Where the cell cycle and histones meet. Genes Dev.

[73] Shen X, Collier JM, Hlaing M, Zhang L, Delshad EH, Bristow J, Bernstein HS (2003). Genome-wide examination of myoblast cell cycle withdrawal during differentiation. Dev Dyn.

[74] Banday AR, Baumgartner M, Seesi SA, Karunakaran DKP, Venkatesh A, Congdon S, Lemoine C, Kilcollins AM, Mandoiu I, Punzo C (2014). Replication-dependent histone genes are actively transcribed in differentiating and aging retinal neurons. Cell Cycle.

[75] Fullwood MJ, Liu MH, Pan YF, Liu J, Xu H, Mohamed YB, Orlov YL, Velkov S, Ho A, Mei PH (2009). An oestrogen-receptor-alpha-bound human chromatin interactome. Nature.

[76] Zhao Z, Tavoosidana G, Sjölinder M, Göndör A, Mariano P, Wang S, Kanduri C, Lezcano M, Sandhu KS, Singh U (2006). Circular chromosome conformation capture (4C) uncovers extensive networks of epigenetically regulated intra- and interchromosomal interactions. Nat Genet.

[77] Maze I, Noh K-M, Soshnev AA, Allis CD (2014). Every amino acid matters: essential contributions of histone variants to mammalian development and disease. Nat Rev Genet.

[78] Maehara K, Harada A, Sato Y, Matsumoto M, Nakayama KI, Kimura H, Ohkawa Y (2015). Tissue-specific expression of histone H3 variants diversified after species separation. Epigenetics Chromatin.

[79] Gonzalez-Sandoval A, Gasser SM (2016). On TADs and LADs: spatial control over gene expression. Trends Genet.

[80] Nie F, Yu X-L, Wang X-G, Tang Y-F, Wang L-L, Ma L (2010). Down-regulation of CacyBP is associated with poor prognosis and the effects on COX-2 expression in breast cancer. Int J Oncol.

[81] Tomás D, Brazáo J, Viegas W, Silva M (2013). Differential effects of high-temperature stress on nuclear topology and transcription of repetitive noncoding and coding rye sequences. Cytogenet Genome Res.

[82] Wittmann M, Queisser G, Eder A, Wiegert JS, Bengtson CP, Hellwig A, Wittum G, Bading H (2009). Synaptic activity induces dramatic changes in the geometry of the cell nucleus: interplay between nuclear structure, histone H3 phosphorylation, and nuclear calcium signaling. J Neurosci.

[83] Nagano T, Lubling Y, Stevens TJ, Schoenfelder S, Yaffe E, Dean W, Laue ED, Tanay A, Fraser P (2013). Single-cell Hi-C reveals cell-to-cell variability in chromosome structure. Nature.

[84] Kauffman SA (1993). The origins of order: self organization and selection in evolution.

[85] Kapranov P, Willingham AT, Gingeras TR (2007). Genome-wide transcription and the implications for genomic organization. Nat Rev Genet.

[86] Mehta IS, Kulashreshtha M, Chakraborty S, Kolthur-Seetharam U, Rao BJ (2013). Chromosome territories reposition during DNA damage-repair response. Genome Biol.

[87] Mehta IS, Amira M, Harvey AJ, Bridger JM (2010). Rapid chromosome territory relocation by nuclear motor activity in response to serum removal in primary human fibroblasts. Genome Biol.

[88] Reddy KL, Zullo JM, Bertolino E, Singh H (2008). Transcriptional repression mediated by repositioning of genes to the nuclear lamina. Nature.

[89] Osborne CS, Chakalova L, Mitchell JA, Horton A, Wood AL, Bolland DJ, Corcoran AE, Fraser P (2007). Myc dynamically and preferentially relocates to a transcription factory occupied by Igh. PLoS Biol.

[90] Nora EP, Lajoie BR, Schulz EG, Giorgetti L, Okamoto I, Servant N, Piolot T, van Berkum NL, Meisig J, Sedat J (2012). Spatial partitioning of the regulatory landscape of the X-inactivation centre. Nature.

[91] Gilbert N, Gilchrist S, Bickmore WA (2005). Chromatin organization in the mammalian nucleus. Int Rev Cytol.

[92] Young MD, Willson TA, Wakefield MJ, Trounson E, Hilton DJ, Blewitt ME, Oshlack A, Majewski IJ (2011). ChIP-seq analysis reveals distinct H3K27me3 profiles that correlate with transcriptional activity. Nucleic Acids Res.

[93] Mastroyiannopoulos NP, Nicolaou P, Anayasa M, Uney JB, Phylactou LA (2012). Down-regulation of myogenin can reverse terminal muscle cell differentiation. PLoS One.

[94] Hjiantoniou E, Anayasa M, Nicolaou P, Bantounas I, Saito M, Iseki S, Uney JB, Phylactou LA (2008). Twist induces reversal of myotube formation. Differentiation.

