# Supplementary material for: Linkages between changes in the 3D organization of the genome and transcription during myotube differentiation in vitro
Source: Skelet Muscle. 2017 Apr 5;7:5. doi: 10.1186/s13395-017-0122-1 (PMC5382473; doi:10.1186/s13395-017-0122-1)
Supplement: Supplementary file 1 — Cell numbers at time of plating and harvesting. Table S2. Alignment summary of RNA-seq reads onto the mouse transcriptome (UCSC-mm10.gtf transcriptome file). Table S3. The number of significantly differentially expressed genes varied between the conditions (FDR-corrected p < 0.05). Table S4. The ten top ranked GO term finder results for the 10% of most significantly upregulated and downregulated genes for the myotubes vs myoblasts comparison (p values are corrected by Bonferroni correction). Table S5. The ten top ranked GO term finder results amongst the top 10% differentially upregulated and downregulated genes between AraC-treated myotubes vs myoblasts (p values are corrected by Bonferroni correction). Table S6. The ten top ranked GO term finder results amongst the top 10% differentially upregulated and downregulated genes between AraC-treated myotubes vs myotubes (p values are corrected by Bonferroni correction). Table S7. HiCUP summary report of total number of valid unique di-tags and the distribution of genome distances which separate the individual reads (tags) from the di-tags. Table S8. Interaction matrices on 500 kb resolution are highly correlated at the level of biological replicates within and between conditions. Table S9. GO terms enriched amongst the genes corresponded to TSS having their PC1 values and transcript levels decreased during the switch from myoblasts to myotubes. Table S10. GO terms enriched amongst the genes corresponded to TSS having their PC1 values and transcript levels decreased during the switch from myoblasts to AraC-treated myotubes. Table S11. GO terms enriched amongst the genes corresponded to TSS having their PC1 values increased during the switch from myoblasts to AraC-treated myotubes. Table S12. GO terms enriched amongst the genes corresponded to TSS having their PC1 values increased during the switch from myotubes to AraC-treated myotubes. (DOCX 43 kb) [file 13395_2017_122_MOESM1_ESM.docx]

| Culture vessel | Time point | myoblasts  (5 × 10^3^/cm^2^) | myotubes*  (2.5 × 10^4^/cm^2^) | AraC treated myotubes*  (+10 µg/mL AraC) |
| --- | --- | --- | --- | --- |
| T-75  Flask  (75 cm^2^) | Plating | 375 000/flask | 1 875 000/flask | 1 875 000/flask |
|  | Harvesting | ~ 5 X 10^6^/flask | >5 X 10^6^/flask | >5 X 10^6^/flask |
| 12W  Plates  (3.9 cm^2^/well) | Plating | 19 500/well | 97 500/well | 97 500/well |
|  | Harvesting | ~ 260 000/well | >260 000/well | >260 000/well |

**Table S1 Cell numbers at time of plating and harvesting.**

* The exact number of myotubes and AraC treated myotubes could not be precisely determined due to the fact that the cells contain multiple nuclei.

| Cell Type | Replicate | | Total reads (pairs) | Aligned reads (pairs) | Concordant pair alignment rate (%) | Multiple alignment rate (%) | Discordant alignment rate (%) | RIN |
| --- | --- | --- | --- | --- | --- | --- | --- | --- |
| myoblasts | | R1 | 7,669,926 | 7,152,323 | 92.9% | 4.5% | 0.4% | 10 |
|  |  | R2 | 7,770,336 | 7,191,114 | 92.2% | 4.8% | 0.4% | 10 |
| myotubes | | R1 | 7,958,568 | 7,398,964 | 92.6% | 5.2% | 0.4% | 9.65 |
|  |  | R2 | 7,972,981 | 7,427,092 | 92.8% | 5.4% | 0.3% | 9.55 |
| AraC treated myotubes | | R1 | 7,934,999 | 7,372,219 | 92.6% | 5.9% | 0.4% | 9.45 |
|  |  | R2 | 7,931,381 | 7,364,568 | 92.5% | 5.6% | 0.4% | 9.55 |

Table S2 Alignment summary of RNA-seq reads onto the mouse transcriptome (UCSC-mm10.gtf transcriptome file).

| Compared cell types | Significantly differentially expressed genes | Up-regulated | Down-regulated |
| --- | --- | --- | --- |
| myotubes vs myoblasts | 5476 | 2700 | 2776 |
| AraC treated myotubes vs myotubes | 2126 | 1040 | 1086 |
| AraC treated myotubes vs myoblasts | 6143 | 2989 | 3154 |

**Table S3 The number of significantly differentially expressed genes varied between the conditions (FDR corrected p<0.05).**

| myotubes vs myoblasts | | | |
| --- | --- | --- | --- |
|  | Gene Ontology term | % of genes from total | Corrected P value |
| Up-regulated (266) | muscle system process | 17.70 | 2.15E-35 |
|  | muscle contraction | 16.20 | 3.09E-35 |
|  | striated muscle cell differentiation | 13.90 | 8.75E-27 |
|  | striated muscle contraction | 10.90 | 2.69E-26 |
|  | myofibril assembly | 7.90 | 1.02E-24 |
|  | muscle cell development | 11.30 | 1.47E-24 |
|  | striated muscle cell development | 10.90 | 1.79E-24 |
|  | muscle structure development | 18.00 | 4.06E-24 |
|  | muscle cell differentiation | 14.30 | 4.01E-22 |
|  | skeletal muscle contraction | 5.30 | 2.32E-16 |
|  |  |  |  |
| Down-regulated (277) | cell cycle | 50.40 | 2.55E-93 |
|  | cell cycle process | 43.20 | 7.65E-86 |
|  | mitotic cell cycle process | 36.30 | 6.12E-83 |
|  | mitotic cell cycle | 36.70 | 6.79E-77 |
|  | nuclear division | 31.30 | 4.06E-74 |
|  | organelle fission | 32.00 | 9.33E-74 |
|  | mitotic nuclear division | 28.40 | 1.64E-72 |
|  | cell division | 29.90 | 5.38E-61 |
|  | chromosome segregation | 20.50 | 2.42E-53 |
|  | single-organism organelle organization | 41.70 | 6.54E-42 |

Table S4 The ten top ranked GO term finder results for the 10% of most significantly up-regulated and down-regulated genes for the myotubes vs myoblasts comparison (p values are corrected by Bonferroni correction).

| AraC treated myotubes vs myoblasts | | | |
| --- | --- | --- | --- |
| Up-regulated (293) | Gene Ontology term | % of genes from  total | Corrected P value |
|  | muscle contraction | 15.00 | 1.12E-34 |
|  | muscle system process | 16.40 | 1.37E-34 |
|  | muscle structure development | 18.80 | 6.54E-29 |
|  | striated muscle cell differentiation | 13.30 | 1.25E-27 |
|  | striated muscle contraction | 10.20 | 1.76E-26 |
|  | striated muscle cell development | 10.60 | 5.87E-26 |
|  | muscle cell development | 10.90 | 6.12E-26 |
|  | myofibril assembly | 7.50 | 1.32E-25 |
|  | muscle cell differentiation | 13.70 | 1.21E-22 |
|  | muscle tissue development | 13.70 | 1.56E-21 |
|  |  |  |  |
| Down-regulated (315) | cell cycle | 36.20 | 4.91E-56 |
|  | mitotic cell cycle process | 25.70 | 4.52E-52 |
|  | cell cycle process | 30.50 | 2.01E-51 |
|  | mitotic cell cycle | 26.30 | 1.27E-48 |
|  | mitotic nuclear division | 20.00 | 1.91E-46 |
|  | nuclear division | 21.90 | 3.15E-46 |
|  | organelle fission | 22.20 | 4.97E-45 |
|  | cell division | 21.90 | 3.41E-40 |
|  | chromosome segregation | 13.70 | 2.44E-31 |
|  | regulation of cell cycle | 20.00 | 5.33E-27 |

Table S5 The ten top ranked GO term finder results amongst the top 10% differentially up-regulated and down-regulated genes between AraC treated myotubes vs myoblasts (p values are corrected by Bonferroni correction).

| AraC treated myotubes vs myotubes | | | |
| --- | --- | --- | --- |
|  | Gene Ontology term | % of genes from  total | Corrected P value |
| Up-regulated(104) | platelet-derived growth factor production | 2.90% | 9.85E-05 |
|  | regulation of platelet-derived growth factor production | 2.90% | 9.85E-05 |
|  | regulation of response to stimulus | 30.50% | 0.00348 |
|  | response to cytokine | 11.40% | 0.00535 |
|  | response to type I interferon | 3.80% | 0.00674 |
|  |  |  |  |
| Down-regulated(108) | developmental process | 54.10% | 3.01E-09 |
|  | single-organism developmental process | 53.20% | 8.21E-09 |
|  | single-organism process | 86.20% | 1.27E-08 |
|  | cellular developmental process | 43.10% | 5.98E-08 |
|  | anatomical structure development | 47.70% | 3.08E-07 |
|  | single-multicellular organism process | 51.40% | 3.33E-07 |
|  | cell differentiation | 40.40% | 3.54E-07 |
|  | single-organism cellular process | 78.00% | 2.46E-06 |
|  | regulation of developmental process | 29.40% | 3.50E-06 |
|  | cell migration | 20.20% | 9.66E-06 |

Table S6 The ten top ranked GO term finder results amongst the top 10% differentially up-regulated and down-regulated genes between AraC treated myotubes vs myotubes (p values are corrected by Bonferroni correction).

|  | myoblasts R1 | myoblasts R2 | myotubes  R1 | myotubes  R2 | AraC treated myotubes  R1 | AraC treated myotubes  R2 |
| --- | --- | --- | --- | --- | --- | --- |
| Unique Di-tags | 25,995,746 | 33,890,861 | 32,591,999 | 45,780,474 | 19,024,490 | 30,112,670 |
| Cis-close  (< 10Kbp) | 704,203 | 939,883 | 958,984 | 1,138,959 | 489,673 | 545,520 |
| Cis-far  (> 10Kbp) | 17,661,476 | 23,026,756 | 19,689,128 | 27,643,216 | 10,381,323 | 14,437,841 |
| Trans | 7,630,067 | 9,924,222 | 11,943,887 | 16,998,299 | 8,153,494 | 15,129,309 |

**Table S7 HiCUP summary report of total number of valid unique di-tags and the distribution of genome distances which separate the individual reads (tags) from the di-tags.**

|  | myoblasts R1 | myoblasts R2 | myotubes  R1 | myotubes  R2 | AraC treated myotubes  R1 | AraC treated myotubes  R2 |
| --- | --- | --- | --- | --- | --- | --- |
| myoblasts  R1 | 1 | 0.8 | 0.75 | 0.75 | 0.72 | 0.75 |
| myoblasts  R2 | 0.8 | 1 | 0.79 | 0.79 | 0.75 | 0.74 |
| myotubes  (Day3) R1 | 0.75 | 0.79 | 1 | 0.93 | 0.88 | 0.89 |
| myotubes  (Day3) R2 | 0.75 | 0.79 | 0.93 | 1 | 0.89 | 0.9 |
| myotubes (Day7+AraC) R1 | 0.72 | 0.75 | 0.88 | 0.89 | 1 | 0.9 |
| myotubes (Day7+AraC) R2 | 0.75 | 0.74 | 0.89 | 0.9 | 0.9 | 1 |

**Table S8 Interaction matrices on 500kb resolution are highly correlated at the level of biological replicates within and between conditions.**

| Gene Ontology term | Cluster frequency | Corrected P-value | Genes annotated to the term |
| --- | --- | --- | --- |
| nucleosome organization | 11 of 90 genes, 12.2% | 7.53E-10 | Hist1h2bb, Hist1h2bc, Hist1h1e, Hist1h4b, Hist1h1c, Hist1h4c, Cebpg, Hist1h2be, Hist1h3c, Hist1h4a, Hist1h1a |
| chromatin assembly or disassembly | 11 of 90 genes, 12.2% | 2.14E-09 | Hist1h2bb, Hist1h2bc, Hist1h1e, Hist1h4b, Hist1h1c, Hist1h4c, Cebpg, Hist1h2be, Hist1h3c, Hist1h4a, Hist1h1a |
| nucleosome assembly | 10 of 90 genes, 11.1% | 2.66E-09 | Hist1h2bb, Hist1h2bc, Hist1h1e, Hist1h4b, Hist1h1c, Hist1h4c, Hist1h2be, Hist1h3c, Hist1h4a, Hist1h1a |
| chromatin assembly | 10 of 90 genes, 11.1% | 1.13E-08 | Hist1h2bb, Hist1h2bc, Hist1h1e, Hist1h4b, Hist1h1c, Hist1h4c, Hist1h2be, Hist1h3c, Hist1h4a, Hist1h1a |
| protein-DNA complex subunit organization | 11 of 90 genes, 12.2% | 4.57E-08 | Hist1h2bb, Hist1h2bc, Hist1h1e, Hist1h4b, Hist1h1c, Hist1h4c, Cebpg, Hist1h2be, Hist1h3c, Hist1h4a, Hist1h1a |

**Table S9 GO terms enriched amongst the genes corresponded to TSS having their PC1 values and transcript levels decreased during the switch from myoblasts to myotubes.**

| Gene Ontology term | Cluster frequency | Corrected P-value |
| --- | --- | --- |
| sulfur compound biosynthetic process | 8 of 152 genes, 5.3% | 0.00017 |
| positive regulation of cellular process | 52 of 152 genes, 34.2% | 0.00627 |
| animal organ development | 40 of 152 genes, 26.3% | 0.00828 |
| organ morphogenesis | 19 of 152 genes, 12.5% | 0.01574 |
| cell migration | 21 of 152 genes, 13.8% | 0.01586 |
| positive regulation of biological process | 55 of 152 genes, 36.2% | 0.01691 |
| locomotion | 24 of 152 genes, 15.8% | 0.02863 |
| regulation of cell migration | 15 of 152 genes, 9.9% | 0.03831 |
| system development | 47 of 152 genes, 30.9% | 0.03917 |

**Table S10 GO terms enriched amongst the genes corresponded to TSS having their PC1 values and transcript levels decreased during the switch from myoblasts to AraC treated myotubes.**

**Table S11 GO terms enriched amongst the genes corresponded to TSS having their PC1 values increased during the switch from myoblasts to AraC treated myotubes.**

| Gene Ontology term | Cluster frequency | Corrected P-value | Genes annotated to the term |
| --- | --- | --- | --- |
| pyroptosis | 3 of 143 genes, 2.1% | 0.00914 | Naip5, Naip2, Naip6 |

| Gene Ontology term | Cluster frequency | Corrected P-value | Genes annotated to the term |
| --- | --- | --- | --- |
| Pyroptosis | 3 of 24 genes, 12.5% | 8.61E-06 | Naip5, Naip2, Naip6 |
| detection of bacterium | 3 of 24 genes, 12.5% | 8.56E-05 | Naip5, Naip2, Naip6 |
| detection of other organism | 3 of 24 genes, 12.5% | 8.56E-05 | Naip5, Naip2, Naip6 |
| detection of external biotic stimulus | 3 of 24 genes, 12.5% | 0.00023 | Naip5, Naip2, Naip6 |
| detection of biotic stimulus | 3 of 24 genes, 12.5% | 0.0003 | Naip5, Naip2, Naip6 |

**Table S12 GO terms enriched amongst the genes corresponded to TSS having their PC1 values increases during the switch from myotubes to AraC treated myotubes.**
