# Supplementary material for: Linkages between changes in the 3D organization of the genome and transcription during myotube differentiation in vitro
Source: Skelet Muscle. 2017 Apr 5;7:5. doi: 10.1186/s13395-017-0122-1 (PMC5382473; doi:10.1186/s13395-017-0122-1)
Supplement: Supplementary file 2 — Supplementary methods. (DOCX 53 kb) [file 13395_2017_122_MOESM2_ESM.docx]

**C2C12 cell culturing**

Myoblasts from the skeletal muscle-derived C2C12 cell line were obtained from American Type Culture Collection [(ATCC® CRL1772™)](http://www.atcc.org/~/ps/CRL-1772.ashx). All experiments were performed using cells at passage 3.

C2C12 myoblasts were propagated at 37 ˚C, 5% CO_2_ in Dulbecco's Modified Eagle Medium (DMEM; high glucose, +pyruvate, +phenol red, +L-Glutamine; Gibco**®** 11995-073) supplemented with 10% fetal bovine serum (FBS; Gibco**®**) and antibiotics (penicillin 100 U/ml, streptomycin 100 μg/ml) (Gibco**®**). Myoblasts were plated at a low cellular density of 5 × 10^3^/cm^2^ (to achieve sub-confluent Myoblast cultures) or a high cellular density of 2.5 × 10^4^/cm^2^ (to allow cell crowding and myogenic differentiation to occur).

Six replicate T75 culture flasks (Greiner bio-one, 658175, 20 mL media volume) were plated for each experimental condition to achieve cell numbers required for HiC. Following 72 hours proliferation, cells which were plated at the low density were harvested as sub-confluent myoblast cultures (myoblasts). At the same time (D0), high density cultures were switched to differentiation media (DMEM, 2% horse serum (HS, Gibco**®** 16050-122), penicillin 100 U/ml, and streptomycin 100 μg/ml) to induce myotube formation. Following 3 days of differentiation myotube cultures were either harvested (myotubes), or switched to differentiation media supplemented with 10 µg/mL Cytosine β-D-arabinofuranoside (AraC, Sigma, C1768) in order to eliminate undifferntiated myoblasts. AraC media was replaced on day 5 before harvesting the AraC treated myotubes on day 7 (AraC treated myotubes). Cell densities at plating and harvesting were as indicated in **(Supplementary Tables S1)**.

**Immunocytochemistry**

At 72 h after proliferation, or 3 and 7 days after switch to differentiation media, culture media from the individual wells of the twelve well plates was removed from Myoblasts, Myotubes, and AraC treated Myotubes respectively and replaced with fresh pre-warmed DMEM supplemented with 10% FBS or 2% HS, myoblasts and myotubes respectively, and MitoTracker® Red CMXRos dye. Cells were incubated (37°C, 30 min) in the presence of MitoTracker^®^ ( final conc. 300 nM in 1 mL DMEM), followed by 2 × 5 min incubations in DMEM (1ml, 37°C) to remove unbound dye. Cells were fixed in formaldehyde/PBS w/v (1ml, final conc. 3.7%, 15 min, 37°C) and washed with three changes of PBS (1ml, 5 min, 37°C each). Fixed cells were permeabilized with Triton X-100/PBS w/v (1ml, final conc. 0.1%) for 10 min at RT, washed three times with 1ml PBS (5min, RT), blocked in 300µl of 1% w/v bovine serum albumin (BSA)/PBS (1 h, RT) and incubated (o/n, 4°C) in blocking buffer containing primary antibody against sarcomeric myosin (MF20 antibody) (300µl, 1:20 dilution). The following day, cells were washed in PBS (5 min, RT) repeated five times, and then incubated in Goat anti-Mouse IgG (H + L) Alexa Fluor® 488 conjugated secondary antibody (300 µl diluted 1:200 in PBS) with 300 nM 4',6-diamidino-2-phenylindole (DAPI; 1 h, RT). Following further washing in PBS (5 min, RT), cells were imaged using the Molecular Devices ImageXpress Micro XLS High content screening system equipped with Andor Zyla CMOS camera and 10x/0.3 NA Plan Fluor lens. Images were captured from nine random pre-selected sites in each of three replicate culture wells. Global linear adjustments to image fluorescent signal brightness/contrast were made in Image J.

**Image capture analysis**

High content screening (HCS) of C2C12 Myoblasts was performed throughout the time-course of differentiation using a Molecular Devices ImageXpress Micro XLS automated wide-field microscope.

MetaXpress software (version 5.3.0.5, Molecular Devices) was used for automated image analysis of the extent of myogenic differentiation. Briefly, the Multi Wavelength Cell Scoring analysis journal was used to quantify the differentiation index (% differentiation) using automated counts of the total number of DAPI stained nuclei per field (DAPI; Wavelength (W)1) and the percentage of W1 counts located within a sarcomeric myosin (MF20)-positive cell body (Alexa Fluor 488; W2). Global linear adjustments to image fluorescent signal brightness/contrast were made to all pixels within an image in Image J software in representative images.

**Preparation of C2C12 HiC libraries**

***Cross-linking of the Myoblast and Myotube cells:*** Cells were washed with 10 mL PBS and the media replaced (20 mL DMEM/serum). For crosslinking, 540 µl of 37% formaldehyde (Sigma-Aldrich) was added to each T-75 flask (1% formaldehyde final conc.) and incubated (10 min, RT, 60 rpm). Unreacted formaldehyde was quenched by addition of glycine (2.5 M; final conc. 0.125 M; 5 min, RT, 60 rpm). Cells were scraped from flasks and transferred to 15 mL falcon tubes, pelleted (800x g, 10 min, 4°C) and the supernatant discarded. Cells were washed with 10 mL PBS (800x g, 10 min, 4°C) and cells from the same Biological Replicate pooled (approximately 20 x 10^6^ myoblasts (four flasks) and 25 x 10 ^6^ myotubes (three flasks). Cross-linked cell pellets were stored at -20°C for not more than two weeks.

***Lysis of cross-linked cells:*** Cross-linked cells were lysed (15 min, on ice) in lysis buffer [(500 μl 10 mM Tris-HCl pH8.0, 10 mM NaCl, 0.2% w/v NP-40) supplemented with 1x EDTA-free protease inhibitors cocktail ((Roche) . Cells were transferred into a Dounce homogenizer, 10 pestle strokes, incubated (on ice, 1 min), before an additional 10 pestle strokes. Cells were transferred to a 1.5 mL microfuge tube, centrifuged (5000 rpm, 2min, RT), and the supernatant discarded. Pelleted chromatin was washed twice (500 μl ice cold 1x NEBuffer 2.1 (NEB)).

***Chromatin digestion:*** Chromatin pellets were suspended in 1mL 1x NEBuffer 2.1 and split into five 210 μl aliquots in 1.5 mL microfuge tubes. NEBuffer 2.1 was added to each tube to 362 μl (final vol.). SDS (38 μl of 1% w/v) was added to each tube, mixedand incubated (10 min, 65°CTubes were transferred onto ice, 44 μl 10% Triton X-100 added and mixed carefully. Chromatin was digested by the addition of 400 Units HindIII (NEB) and incubation (37°C, with rotation, 18 hrs).

***Restriction site filling and biotin labelling:*** Following digestion, chromatin was placed on ice. One of the five tubes served as a control and was kept separate from the remaining four tubes which were prepared for blunt end ligation.

Digested restriction sites were marked by incorporating biotinylated dCTP into the overhangs. Nucleotide triphosphates (1.5 μl 10 mM dATP, 1.5 μl 10 mM dGTP, 1.5 μl 10 mM dTTP,37.5 μl 0.4 mM biotin-14-dCTP (Invitrogen)) and 10 μl 5U/μl Polymerase I, Large Klenow (NEB) were added to each of the four tubes that were being used for HiC library preparation. Tubes were incubated (37°C, 45 min, with rotation) and transferred onto ice.

All reactions were inactivated by the addition of 86 μl 10% SDS and incubation (65°C, 30 min) and placed on ice.

***Blunt end DNA ligation:*** DNA ligation was performed in dilute conditions. Five aliquots of 7.61 ml ligation mix were prepared [745 μl 10% Triton X-100, 745 μl 10x ligation buffer (500 mM Tris-HCl pH7.5, 100 mM MgCl2, 100 mM DTT), 80 μl 10 mg/ml BSA, 80 μl 100 mM ATP and 5.96 ml water] and added to five 15 ml Sarstedt tubes. Chromatin (including the 3C control) was transferred to 15 ml tubes. For ligation of the 3C control, 10 μl 1U/μl T4 DNA ligase (Invitrogen) was added to the 3C tube. For blunt-end ligation, 50 μl 1U/μl T4 DNA ligase (Invitrogen) was added to each of the remaining four tubes, to compensate for the lower efficiency of the blunt-end ligation reaction. All five tubes were incubated (16°C, 4 hrs, with inversion every 30 min).

***Reversal of the cross-links and DNA purification:*** Proteinase K (20 mg/ml; 25 µl) was added and the ligation mixtures incubated at 65°C overnight. Samples were spiked with an additional 25 μl of proteinase K and incubated for a further 2 hrs (65°C). Reactions were incubated at RT for 30 min and transferred to five 50 ml conical tubes.

Phenol (10 ml, pH 8.0) was added to each tube, vortexed (2 min) and centrifuged (3,500 rpm, 10 min). Supernatants were transferred to five new 50 ml conical tubes before another round of phenol extraction. Supernatants were transferred to five 35 ml centrifugation tubes and the volumes in the tubes increased to 10 ml per tube with 10 mM Tris pH8.0, 1 mM EDTA (1x TE). DNA was precipitated by addition of 3M Na-acetate (1/10 v/v) and ice-cold 100% ethanol (2.5 v/v). Tubes were inverted several times and incubated at -80°C for two hours. Tubes were centrifuged (10,000x g, 4°C, 40 min), supernatant discarded, DNA pellets dissolved in 450 μl 1x TE buffer, and transferred to 1.7 ml microfuge tubes.

Two DNA extractions were performed to increase the purity of the DNA. To the contents of the 1.7 mL tubes, 500 μl phenol pH 8.0:chloroform (1:1) was added, vortexed (30 seconds) and centrifuged (14000 rpm, 5 min, RT). Supernatants (each ~400 μl) were transferred to five new 2 ml tubes before the addition of 40 μl 3M Na-acetate, and mixing by inversion. Ethanol (1 ml, 100%) was added to each sample, tubes inverted, incubated (- 80°C, 45 min), and centrifuged (12000 x g, 20 min, 4°C). The supernatant was discarded and pellets washed once with 500 μl 70% ethanol. Pellets were briefly air-dried and suspended in 25 μl 1x TE. RNAse A (1 μl 1 mg/ml) was added and samples incubated (37°C for 15 min). The contents of the four tubes that underwent restriction site end filling and biotin labelling were pooled together to form a complete HiC library.

***Removal of biotin from unligated ends:*** The removal of the biotin from unligated ends was achieved using T4 DNA polymerase. Four reactions per HiC library were set up, each of which contained ~ 5 μg DNA (estimated by visualizing 2 and 6 µl aliquots of the purified DNA libraries following separation on a 2% (w/v) agarose gel (90V/30min)). To each HiC library aliquot; 1 μl 10 mg/ml BSA, 10 μl 10x NEBuffer 2.1, 1 μl 10 mM dATP, 1 μl 10 mM dGTP and 5 Units T4 DNA polymerase (NEB) was added and the total volume made up to 100 μl with 1x TE. Samples were incubated (12°C, 2h) and reactions stopped by the addition of 2 μl 0.5 M EDTA pH8.0. Finally, DNA was purified by one phenol pH8.0:chloroform (1:1) extraction followed by ethanol precipitation. Purified DNA pellets were suspended in 100 μl water.

***Shearing of the HiC libraries and repair of the sheared DNA ends:*** Long DNA fragments in the HiC libraries were fragmented (EpiShear™ Cooled Sonication Platform, Active Motif) to achieve optimal sequencing sizes of between 150 – 800 bp. Fragmentation was achieved by sonication (6 min run time, 59 sec/on, 59 sec/off, 4°C) in polystyrene sonication tubes.

Nicks in fragments were repaired by addition of 14 μl 10x ligation buffer (as for blunt-end ligation), 14 μl 2.5 mM dNTP mix, 5 μl T4 DNA polymerase (NEB), 5 μl T4 polynucleotide kinase (NEB), 1 μl Klenow DNA polymerase (NEB) and 1 μl water. Samples were incubated at RT for 30 min.

DNA was purified using Qiagen MinElute columns (Qiagen) according to the manufacturer’s instructions. DNA was eluted in 2 x 15 μl Tris-Low-EDTA buffer TLE (10 mM Tris pH8.0, 0.1 mM EDTA).

***A-tailing of the blunt DNA fragments:*** An 'A' was added to the 3' ends of the end repaired blunt DNA fragments to prevent them from ligating to one another during adapter ligation. The tailing reaction (37°C for 30 min) was carried out following addition of 5 μl 10x NEBuffer2.1, 10 μl 1 mM dATP, 2 μl water and 3 μl Klenow (exo-) (NEB) to each of the HiC libraries. The reaction was stopped (65°C, 20 min) and cooled on ice before reducing the volume to 20 μl with a speedvac Express SC250EXP SpeedVac™ Concentrator System (Thermo Scientific; 1h, 55 °C, pressure(2)).

***Size selection:*** DNA was separated by electrophoresis (1.5% w/v agarose gel, 1x TAE, 1.5 hrs, 80 V). The gel was stained with SYBR green (Invitrogen), visualized (Gel Doc™ EZ Imaging System; Bio-Rad). DNA between 300 and 650 base pairs was excised and purified using the Qiaquick Gel Extraction Kit (according to manufacturer’s recommendation). Size selected DNA was eluted in 2x 50 μl 10mM TLE buffer and made up to 300 μl with TLE.

DNA concentration was measured using the Quant-iT™ dsDNA High-Sensitivity (HS) Assay Kit (Invitrogen) and a Qubit® 3.0 Fluorometer (Invitrogen). The HiC libraries contained between 263-630 ng of DNA: (Myoblasts Replicate 1 (684 ng), Myoblasts Replicate 2 (309.6 ng), Myotubes Replicate 1 (263.9 ng), Myotubes Replicate 2 (630 ng), AraC treated Myotubes Replicate 1 (311 ng), AraC treated Myotubes Replicate 2 (444.6 ng)).

***Biotin pull-down:*** Dynabeads MyOne Streptavidin C1 Beads (Invitrogen) were used to pull-down the biotin labelled fragments. All subsequent steps were performed in DNA LoBind tubes (Eppendorf). Streptavidin beads (100 μl) were washed twice (3 min, RT with rotation) with 400 μl Tween Wash Buffer (TWB; 5 mM Tris-HCl pH8.0, 0.5 mM EDTA, 1 M NaCl, 0.05% Tween). Beads were reclaimed by holding against a 96S Super Magnet Plate (Alpaqua) for 1 min and the supernatant was removed. All subsequent washing steps were performed using this procedure.

Washed beads were resuspended in 300 μl 2x Binding Buffer (BB) (10 mM Tris- HCl pH8.0, 1 mM EDTA, 2 M NaCl) and combined with 300 μl HiC DNA to make up the final concentration of 1x BB buffer. The mixture was incubated (RT, 15 min, with rotation) before the beads were reclaimed (as above). The Streptavidin beads were resuspended in 400 μl 1x BB and transferred to a new tube. The beads were then reclaimed and resuspended in 100 μl 1x ligation buffer, transferred to a new tube and reclaimed, before being suspended in 50 μl 1x ligation buffer.

***Preparation of the HiC libraries for Paired End sequencing:*** Paired end sequencing adapters were ligated onto the HiC libraries (Truseq nano DNA LT kit; Illumina). A 2.5 μl aliquot of the thawed unique adapter index, 2.5 μl resuspension buffer and 2.5 μl Ligation Mix 2 was added to each HiC library and the mixture incubated (2 hr, 30°C). Stop Ligation buffer (5 μl) was added to each reaction and fragments that were ligated to the adapters purified by magnet. Adapter ligated DNA was washed with 400 μl of 1x TWB. Beads were resuspended in 400 μl 1x TWB and the mixture transferred to a new tube. Streptavidin beads were reclaimed by washing with: 1) 200 μl 1x BB; 2) 200 μl 1x NEBuffer 2.1; and 3) 50 μl 1x NEBuffer 2.1. The resulting beads were suspended in 50 μl 1x NEBuffer 2.1 and transferred to a new tube.

For HiC library amplification, two PCR reactions (22.5 μl template [2.5 μl Streptavidin beads and 20 μl 1XNEBbuffer] + 22.5 μl Enhanced PCR mix + 5 μl Primer cocktail) were set up to provide sufficient PCR products for sequencing for all samples with the exception of Myoblasts Replicate 2 and AraC treated Myotubes Replicate 1 where 4 PCR reactions were used. The PCR program was (95°C, 3 min; 15 X [98°C, 20 s; 60°C, 15 s; 72°C, 30 s]; 72°C, 5 min).

PCR products, from each HiC library, were purified with 1x volume Ampure beads (Truseq nano DNA LT kit; Illumina). PCR bound Ampure beads were separated using a magnet (2 min, RT) and the supernatant discarded. HiC library bound Ampure beads were washed twice with 200 μl of 80% ethanol while the tube remained against the magnet. The beads were air-dried (6 min, RT) and the DNA eluted by suspending the beads in 50 μl TLE (10 mM Tris pH8.0, 0.1 mM EDTA). The size distribution and purity of the cleaned amplified HiC libraries was measured by running duplicate 1 μl aliquots on a 2100 Bioanalyzer Instrument (Agilent) using the Agilent DNA 12000 Kit (Agilent).

Finally, ~ 1μg of amplified and cleaned PCR products from each HiC library was sent to BGI (Beijing Genomics Institute) for paired end sequencing using the Illumina (HiSeq 2500) platform. Between 188 X 10^6^ and 292 X 10^6^ paired 150 bp reads were sequenced per library. Greater than 97% of the reads had Phred quality scores ≥ 20.

***Mapping of the HiC libraries and generation of QC reports***

The HiCUP (hicup_v0.5.3; <http://www.bioinformatics.babraham.ac.uk/projects/hicup/>) pipeline was used to analyse the HiC libraries. Sequencing reads were mapped to the reference genome (Mus_musculus_GRCh38) using bowtie aligner to generate BAM files.

The HOMER HiC software pipeline (http://biowhat.ucsd.edu/homer/)[1] was used to generate interaction matrices, to identify of A and B compartments, and to determine significant interactions. Briefly, paired-end (*i.e*. di-tags) reads outputted from HiCUP were checked for GC content (“-checkGC”). Reads originating from regions with unusually high tag density “5 fold higher” (‘‘-removeSpikes 10000 5’’) were removed. Read pairs were filtered for distance from the HindIII restriction site and only pairs where both ends mapped near HindIII restriction sites (*e.g.* maximum distance from restriction site is 1.5x of the insert fragment size) were retained. Normalized interaction matrices for each biological replicate were generated using 500kb bin size resolution and the “-corr” function which replaces the number of interactions with a Pearson Correlation Coefficient representing how each region interacts with any other region. This adds transitive information to the problem. For example, if two regions are interacting with the same loci on the interaction matrix the assumption is made that they are probably similar themselves and will have high Pearson Correlation Coefficient (*i.e.* similar to 1) and if two regions are interacting with different loci they will have negative correlation coefficient. Following confirmation that the normalized interaction matrices (500kb resolution) were highly correlated between the biological replicates; subsequent analysis were performed on the data for the combined biological replicates to compensate for the relatively low number of di-tags of the individual replicates.

***Identification of compartments:*** A and B compartments were identified, for each condition, using interaction matrices with resolution at 400kb and a “-superRes” of 500kb. A and B compartment identification was performed using the runHiCpca.pl script within HOMER. This method follows a standardised method introduced by Dekker’s lab [2]. In brief the steps for the identification of compartments were as follows: 1) normalized interaction matrices were generated; 2) correlation between contact profiles from each region against each other region was calculated; 3) Principle components were computed from the correlation matrix for each chromosome; and 4) PC1 and PC2 values for each region were output in separate files. Regions which had expected reads <3, or sequencing depth < 0.15 fraction of mean, or sequencing depth > 4 standard deviation were removed from the analyses. The direction of the Eigenvalues is arbitrary and therefore positive values were set to “A” and negative values were set to “B” based on their association with transcription start sites (TSS).

***Comparison of the interaction profiles between conditions:*** To compare the interaction profiles between conditions, the “getHiCcorrDiff.pl” script in HOMER was used with parameters “res 400000” “superRes 500000”. This script undertakes a direct comparison of matrices generated with the “corr” function between two different experiments. If the interaction profile of a locus in one experiment is similar to the interaction profile of that same locus in another experiment the correlation will be high, if the locus interacts with different regions in the two experiments, the correlation will be low.

Regions that were negatively correlated between the conditions were selected for further analyses. The TSS belonging to these regions were identified using “**annotatePeaks.pl”** simultaneously with the identification of the distribution of PC1 values across the TSS, in 500 bp windows upstream and downstream to the TSS. The transcript levels of the genes corresponding to the TSS were pulled out from the transcriptome data. Genes that had undetectable transcript levels in both conditions under comparison were discarded from the gene group. A Wilcoxon signed-rank test was used to determine if the population mean ranks differs for PC1 values and transcript levels in transition from one experiment to the other for each group. The distribution of the PC1 values and transcript levels was plotted using R (“barplot” function)[3].

The list of genes within which had their PC1 values reduced or increased from one experiment to another and had detectable transcript levels in at least one of the compared conditions were subjected to term enrichment analysis for “biological process” using GOTermFinder (http://go.princeton.edu/cgi- bin/GOTermFinder)[4]. The p-value cut-off was set at 0.05 and gene lists queried against the Mouse Genome Informatics database (MGI).

***Screening of differentially expressed genes for their compartment membership:*** “annotatePeaks.pl” was used with the “hist” option. This generated histograms of PC1 values in 500kbp regions (1000 bp bins) surrounding the TSSs of genes that fell within the top10% and bottom 10% differentially expressed transcripts between the different conditions.

***Identification of TADs:*** Consensus non-overlapping domains (TADs) were identified using the “armatus” algorithm with parameters “g 1.0 -s 0.05 -r 400000” [5]. The input for domain identification was the normalized matrices generated by HOMER with “-res 400kb” and “-superRes 500kb”.

***Identification of significant interactions:*** Significant interactions were identified for each condition at 400kbp resolution, without specifying super-resolution. For two given loci that could potentially interact, HOMER models their randomly expected read counts using the cumulative binomial distribution, such that the total number of trials is the number of reads that could possibly map between the loci, the rate of success is the expected interaction frequency, and the number of observed successes is the number of observed reads mapping between the loci. Interactions which had p value < 0.001 and False Detection rate (fdr) <=0.01 were deemed as significant for each condition.

Venn diagrams were plotted using R (“Vennerable” package)[3].

***Pairwise feature enrichment determination at the endpoints of interactions:*** Published Chip-seq data were mined to obtain Chip enrichment data for PolII, H3K4me2, H3K4me3, H3K27me3 in C2C12 Myoblasts and Myotubes respectively [6]. Chromosome coordinates from the earlier genome version (mm9) were converted to coordinates for the *Mus musculus* (mm10 genome version) using LiſtOver [7]. Myotubes in the Chip-seq experiments were harvested at Day 4 after a medium switch which is similar to the culturing conditions used to isolate our Day3 myotubes. We also used the Chip-seq data for myotubes harvested at Day4 to test for interaction pairwise enrichment features in the AraC treated myotubes on the basis that the morphology, HiC interaction profiles and transcriptomes of the myotubes and AraC treated myotubes were highly similar. For the calculation of enrichment ratios, the peaks for the epigenetic marks and the groups of genes which were tested were linked to the interaction resolution (400kbp) using the “mergePeaks” –d 400000” command.

***RNA extraction***

Myoblasts, Myotubes and AraC treated myotubes were grown on 12 well plates in two biological replicates. Cells in each of nine wells were treated with 390 μl Trizol (Invitrogen; 5 min, RT). Cell contents were homogenized by pipetting to dissociate the nucleoprotein complexes, before transferring the content from each well to a new 1.5 mL microfuge tube. Homogenized RNA samples were stored at -80°C and the RNA extracted within one month of the date of storage.

RNA extraction continued with the addition of 58 μl chloroform to each tube. Tubes were inverted, incubated (RT for 2 min), and centrifuged (12000x g, 15 min, 4°C). The upper aqueous layer (~150 μl) was transferred to a new tube and 150 μl ethanol (70% v/v in DEPC-treated water) added. Samples were mixed by inversion and the extraction continued using the RNAeasy Mini Kit (Qiagen) spin columns (according to the manufacturer’s instructions). For the RNA elution 2 x 15 μl RNAase-free water was added to the center of the Spin Cartridge Column and incubated (1 min, RT) before the RNA was retrieved by a final centrifugation (12000x g, 30 sec, RT).

***Transcriptome analysis of the reference data set***

To validate the differential expression of myogenic molecular markers in our RNA-seq data, the transcript levels of 13 genes were analysed and compared to an independent RNA-seq data set generated from proliferating C2C12 myoblasts (24 h after plating), Myotubes at 60 h (2.5 Days), and myotubes after 7 days of differentiation [8] (Supplementary methods).

Trapnell *et al*. isolated total RNA from proliferating C2C12 myoblasts (24 h after plating), Myotubes at 60 h (2.5 Days), and myotubes after 7 days of differentiation. The Day7 myotubes in Trapnell *et al.* were not exposed to AraC [8].Briefly, sequenced RNA reads described from Trapnell and colleagues [8] were downloaded from GSE20846 and quality checked (http://www.bioinformatics.babraham.ac.uk/projects/fastqc/). The reverse reads had low average quality score 4>x>23 and were excluded from further analysis. The forward reads were trimmed using prinseq (prinseq v0.20.4-lite) to include only sequences with a Phred Quality score >20. Sequences were kept if the total read length remained > 40 bases after trimming. The average sequence quality score for the forward reads after trimming was 21>x>31. The total number of sequencing reads used for mapping to the reference genome was between 11.2 x10^6^ >x>17.8 x10^6^. Further mapping and differential gene expression analyses were performed as described for the experimental data sets

**Supplementary references**

1. Heinz S, Benner C, Spann N, Bertolino E, Lin YC, Laslo P, et al. Simple Combinations of Lineage-Determining Transcription Factors Prime cis-Regulatory Elements Required for Macrophage and B Cell Identities. Mol. Cell [Internet]. 2010;38:576–89. Available from: http://linkinghub.elsevier.com/retrieve/pii/S1097276510003667

2. Lieberman-Aiden E, van Berkum NL, Williams L, Imakaev M, Ragoczy T, Telling A, et al. Comprehensive mapping of long-range interactions reveals folding principles of the human genome. Science [Internet]. 2009 [cited 2014 Jan 21];326:289–93. Available from: http://www.pubmedcentral.nih.gov/articlerender.fcgi?artid=2858594&tool=pmcentrez&rendertype=abstract

3. R Core Team. R: A Language and Environment for Statistical Computing [Internet]. Vienna, Austria; 2013. Available from: http://www.r-project.org/

4. Boyle EI, Weng S, Gollub J, Jin H, Botstein D, Cherry JM, et al. GO::TermFinder--open source software for accessing Gene Ontology information and finding significantly enriched Gene Ontology terms associated with a list of genes. Bioinformatics [Internet]. 2004;20:3710–5. Available from: http://bioinformatics.oxfordjournals.org/cgi/doi/10.1093/bioinformatics/bth456

5. Filippova D, Patro R, Duggal G, Kingsford C. Identification of alternative topological domains in chromatin. Algorithms Mol. Biol. [Internet]. 2014;9:14. Available from: http://almob.biomedcentral.com/articles/10.1186/1748-7188-9-14

6. Asp P, Blum R, Vethantham V, Parisi F, Micsinai M, Cheng J, et al. Genome-wide remodeling of the epigenetic landscape during myogenic differentiation. Proc. Natl. Acad. Sci. 2011;108:E149–58.

7. Hinrichs AS, Raney BJ, Speir ML, Rhead B, Casper J, Karolchik D, et al. UCSC Data Integrator and Variant Annotation Integrator. Bioinformatics. 2016;btv766-.

8. Trapnell C, Williams B a, Pertea G, Mortazavi A, Kwan G, van Baren MJ, et al. Transcript assembly and quantification by RNA-Seq reveals unannotated transcripts and isoform switching during cell differentiation. Nat. Biotechnol. Nature Publishing Group; 2010;28:511–5.
