# Supplementary material for: Linkages between changes in the 3D organization of the genome and transcription during myotube differentiation in vitro
Source: Skelet Muscle. 2017 Apr 5;7:5. doi: 10.1186/s13395-017-0122-1 (PMC5382473; doi:10.1186/s13395-017-0122-1)
Supplement: Supplementary file 3 — Determination of the total nuclei and percent of differentiation in the course of cell culture. Figure S2. Transcript levels, measured as FPKM values, were highly correlated between biological replicates. Figure S3. Cytosolic DNA-sensing pathway was enriched (p = 0.049) within the gene set containing the top 10% of significantly upregulated genes for AraC-treated myotubes vs myotubes comparison. Figure S4. QC report generated after processing of the myoblasts replicate 1 Hi-C library. Figure S5. QC report generated after processing of the myoblasts replicate 2 Hi-C library. Figure S6. QC report generated after processing of the myotubes (day 3) replicate 1 Hi-C library. Figure S7. QC report generated after processing of the myotubes (day 3) replicate 2 Hi-C library. Figure S8. QC report generated after processing of the myotubes (day 7 + AraC) replicate 1 Hi-C library. Figure S9. QC report generated after processing of the myotubes (day 7 + AraC) replicate 2 Hi-C library. Figure S10. Patches of replication-dependent histone variants spatially clustered within the HIST1 locus. Figure S11. Distributions of shared and unique interactions and TADs across the three conditions. Figure S12. Two consensus TADs visualized on the UCSC genome browser spanning developmentally regulated arrays of muscle genes and their corresponding histone mark signals on chr1. (DOCX 2373 kb) [file 13395_2017_122_MOESM3_ESM.docx]

|   Figure S1 Determination of the total nuclei and % of differentiation in the course of cell culture.  The total number of nuclei decreased during the course of myogenic progression (A), while the percentage of differentiation gradually increased (B). For determination of the percentage of differentiation the total number of DAPI stained nuclei were counted and the percentage of total nuclei located within myosin positive cell body was determined. The counts for total nuclei and percentage of differentiation were performed from nine random preselected sites in triplicates for each condition. Total nuclei count and % differentiation are plotted as Mean ± SEM as averaged from the two biological replicates. Letters on the error bars indicate the significant differences between time-points as determined by one-way ANOVA analysis (p < 0.05). |
| --- |

|   Figure S2 Transcript levels, measured as FPKM values, were highly correlated between biological replicates.  Transcript levels were plotted as scatterplots for replicates 1and 2: A) Myoblasts - R^2^=0.98; B) Myotubes(Day3) - R^2^=0,97; C) Myotubes(Day7+AraC) - R^2^=0.97 (p<0.001 for all conditions). R1 (Replicate 1) and R2 (Replicate 2) of the corresponding conditions. |
| --- |


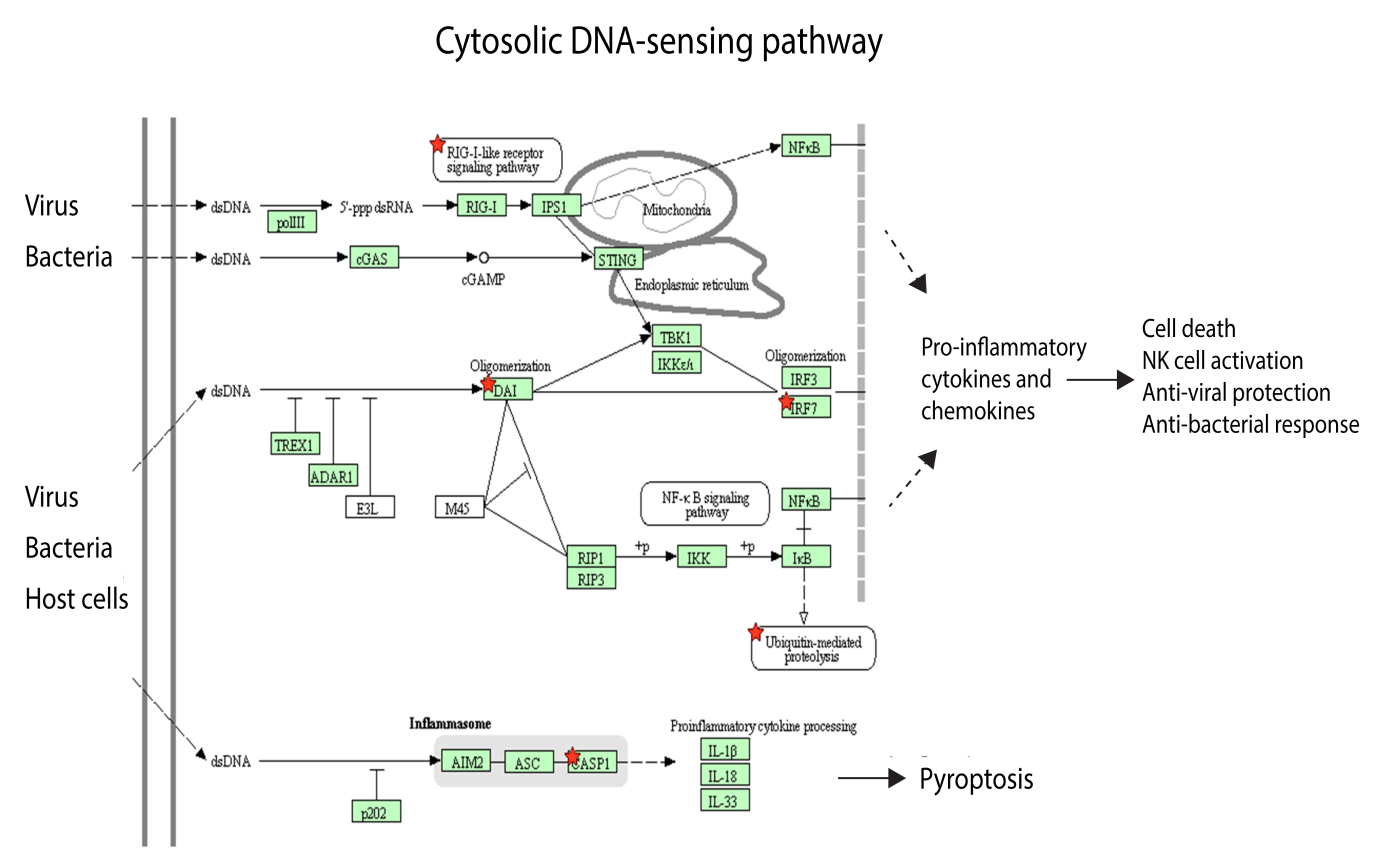


**Figure S3. Cytosolic DNA-sensing pathway was enriched (p=0.049) within the gene set containing the top10% of significantly up-regulated genes for AraC treated myotubes vs myotubes comparison.**

The genes were queried against KEGG pathways. Genes marked with red stars are the overlapping genes between the queried subset and the genes involved in the pathway.

| 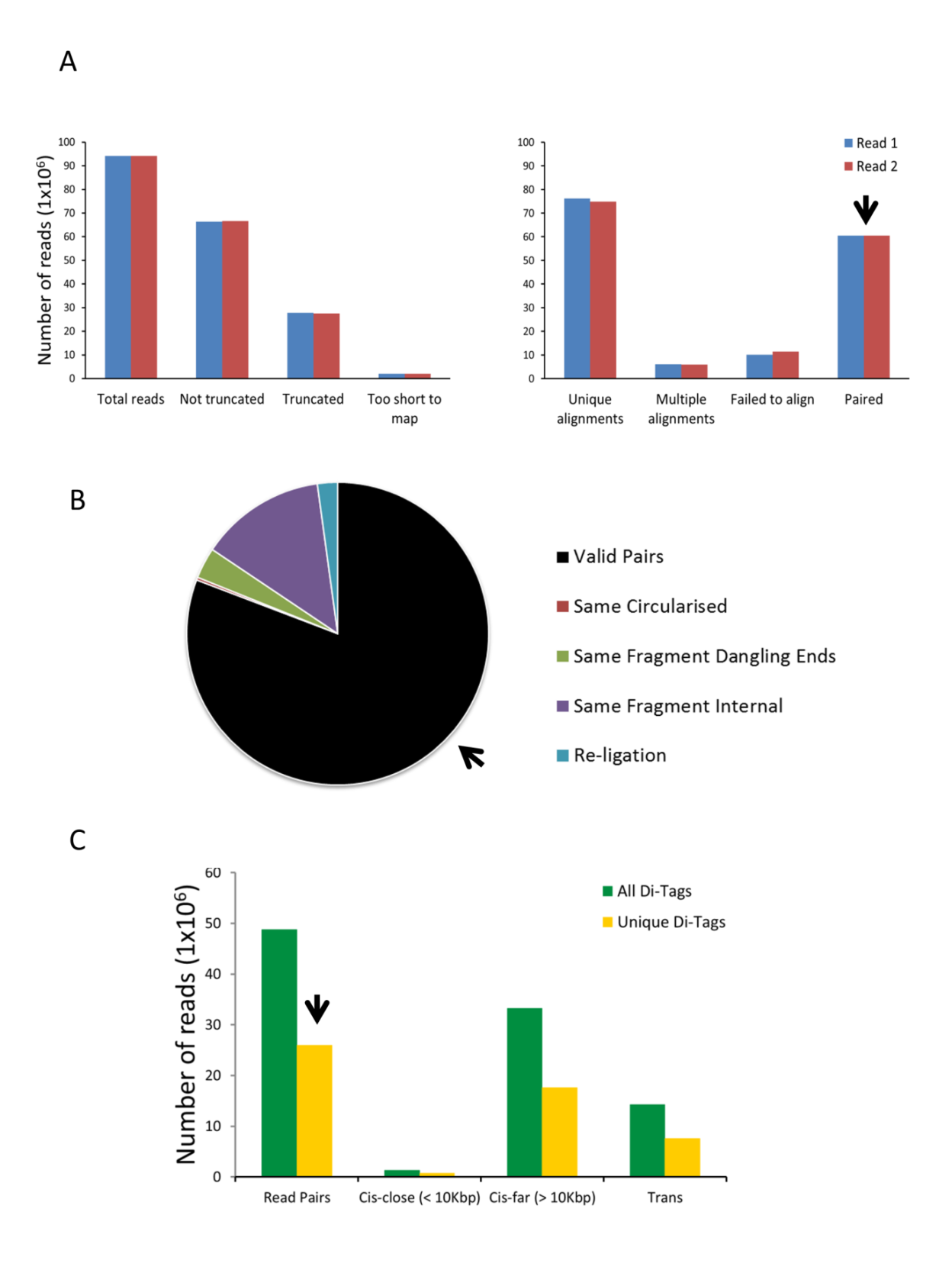  **Figure S4 QC report generated after processing of the Myoblasts Replicate 1 HiC library.**  64.1% (A) of the reads mapped as a valid pair to reference genome, 80.8% (B) of the mapped reads passed the filtering step and 25.9 million reads (C) remained after the de-duplication. |
| --- |

| 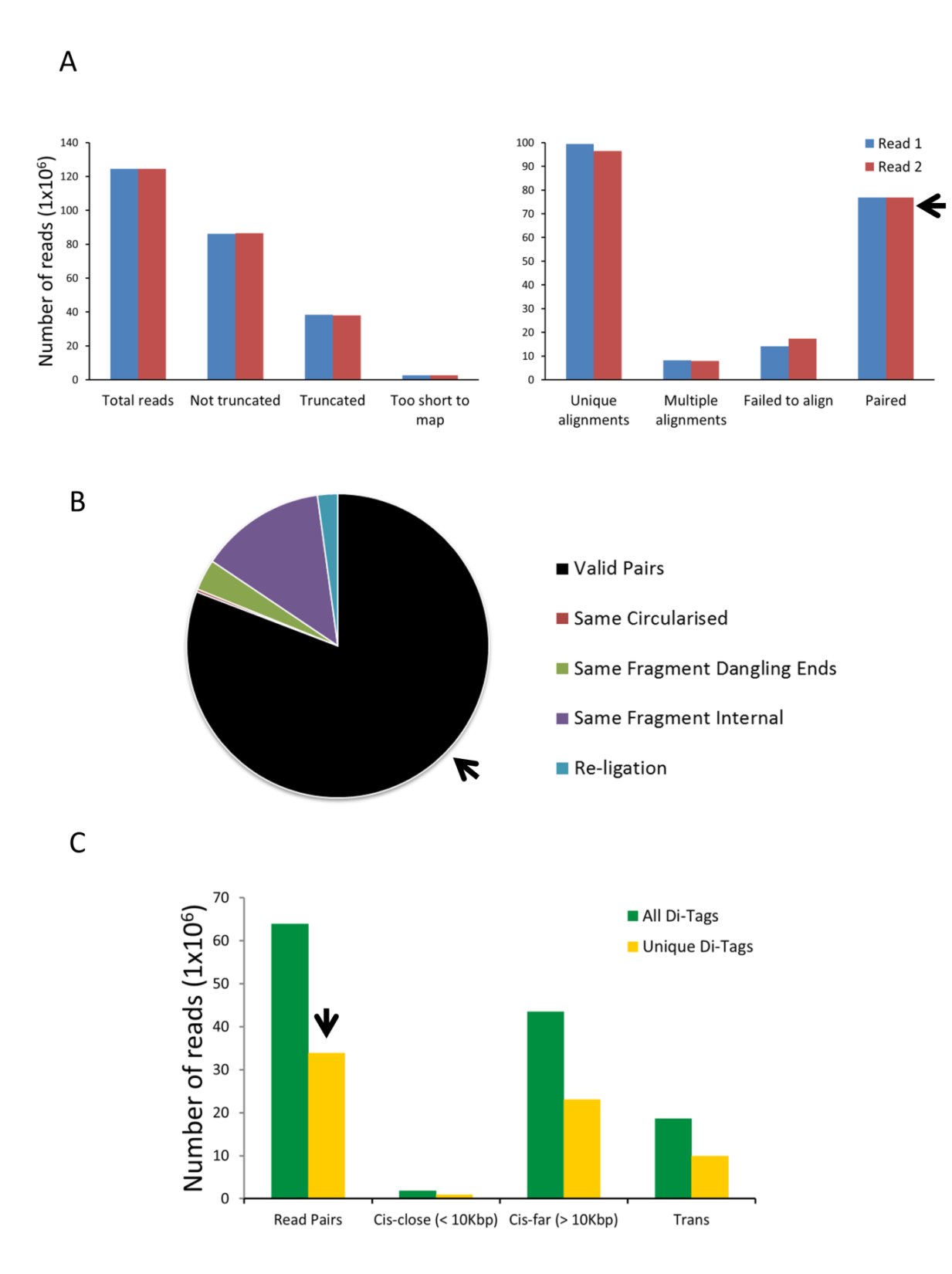  **Figure S5 QC report generated after processing of the Myoblasts Replicate 2 HiC library.**  61.8% (A) of the reads mapped as a valid pair to reference genome, 83.1% (B) of the mapped reads passed the filtering step. 33.8 million reads (C) remained after the de-duplication. |
| --- |

| 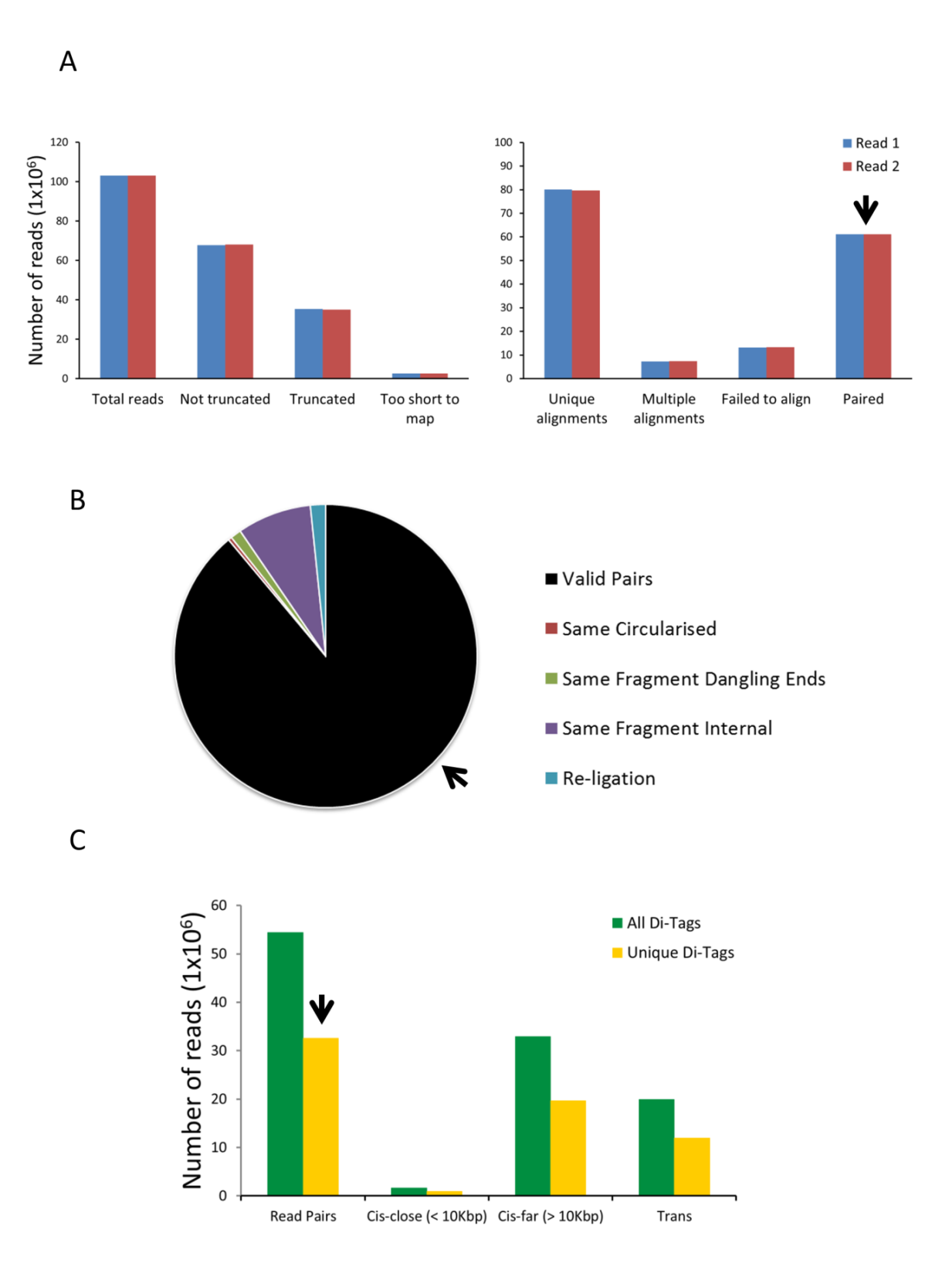  **Figure S6 QC report generated after processing of the Myotubes(Day3) Replicate 1 HiC library.**  59.4% (A) of the reads mapped as a valid pair to reference genome, 88.9% (B) of the mapped reads passed the filtering step. 32.5 million reads (C) remained after the de-duplication. |
| --- |

| 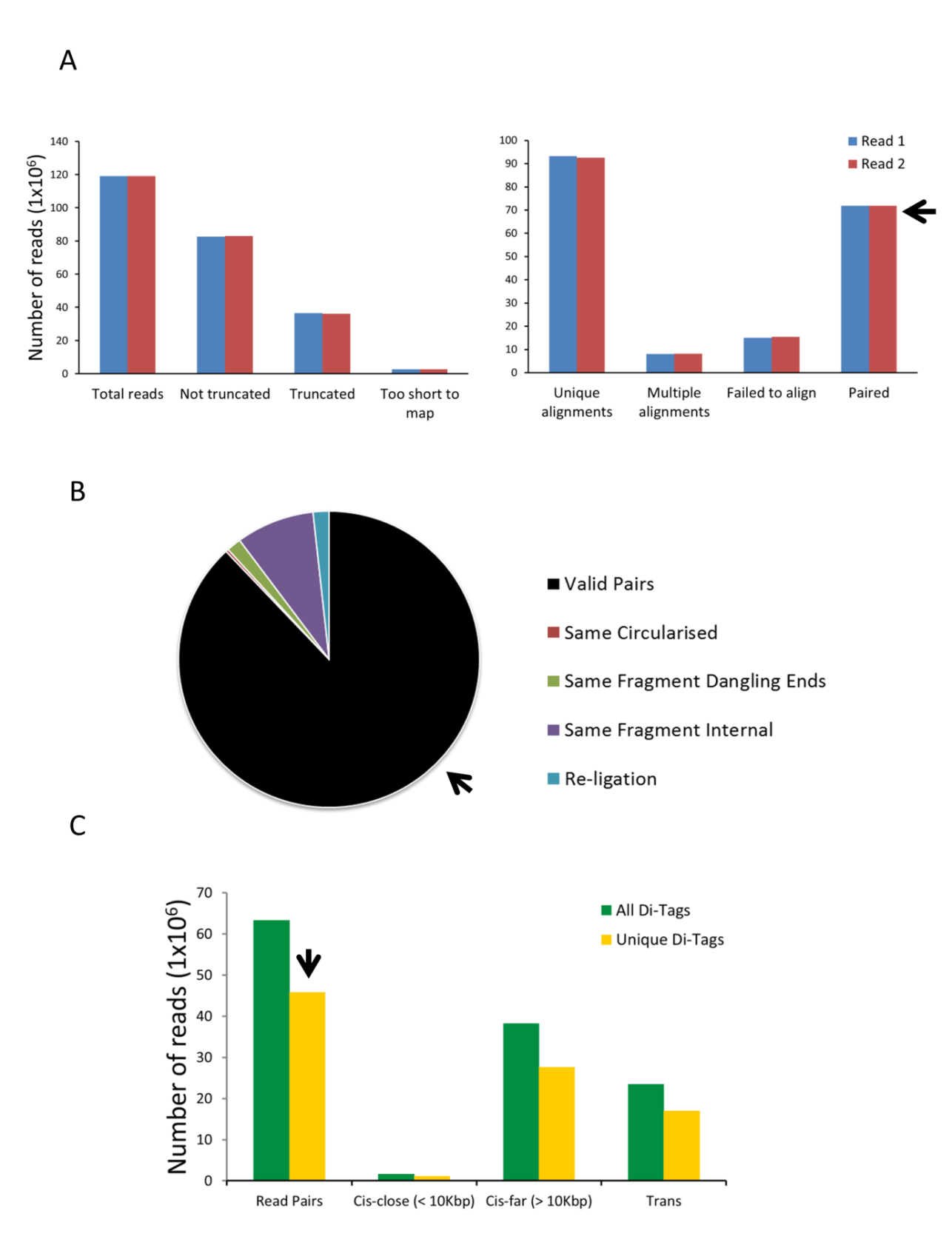  **Figure S7 QC report generated after processing of the Myotubes(Day3) Replicate 2 HiC library.**  60.4% (A) of the reads mapped as a valid pair to reference genome, 88% (B) of the mapped reads passed the filtering step. 45.78 million reads (C) remained after the de-duplication. |
| --- |

| 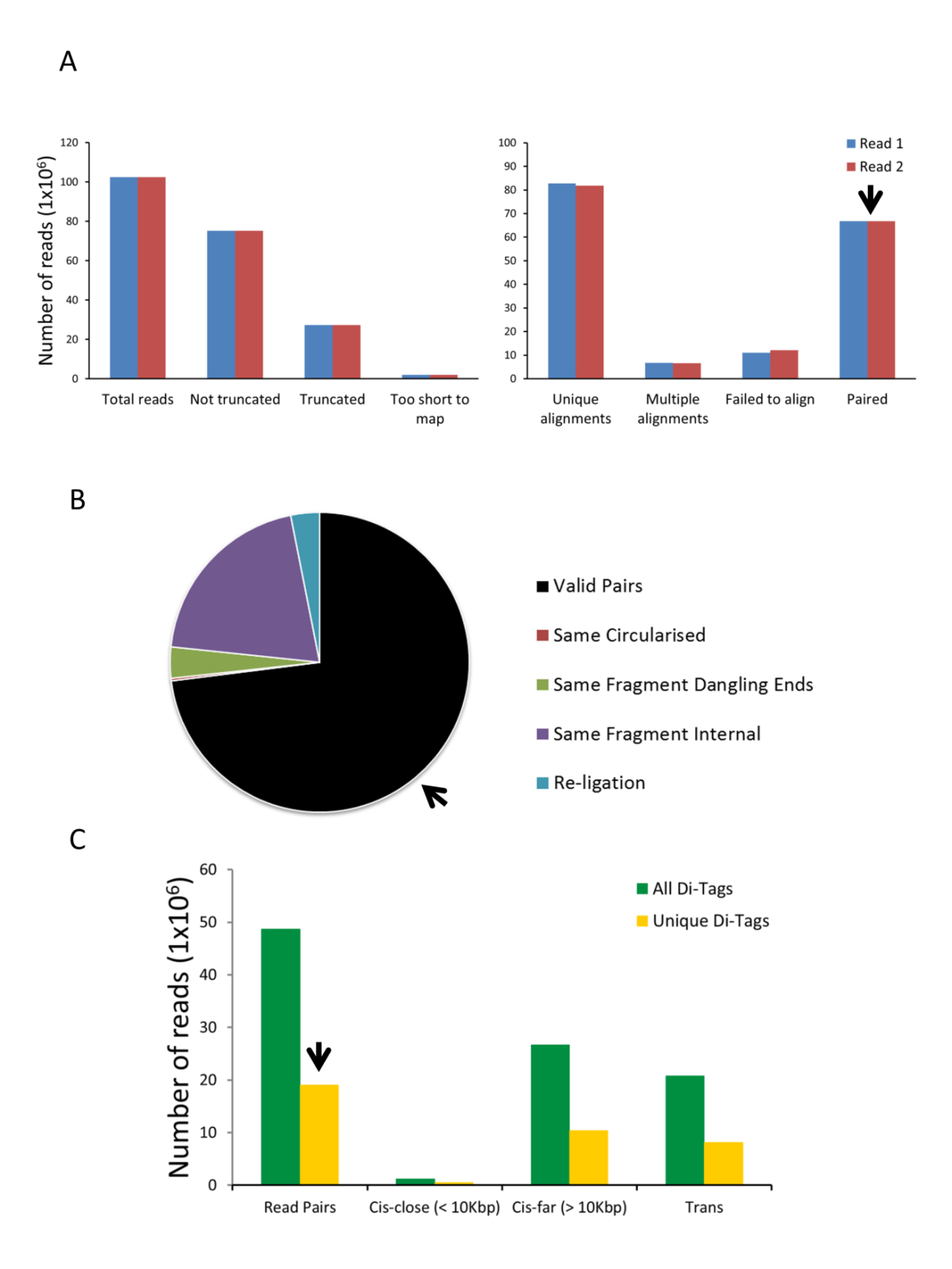  Figure S8 QC report generated after processing of the Myotubes(Day7+AraC) Replicate 1 HiC library.  62.5% (A) of the reads mapped as a valid pair to reference genome, 73% (B) of the mapped reads passed the filtering step. 19 million reads (C) remained after the de-duplication. |
| --- |

| 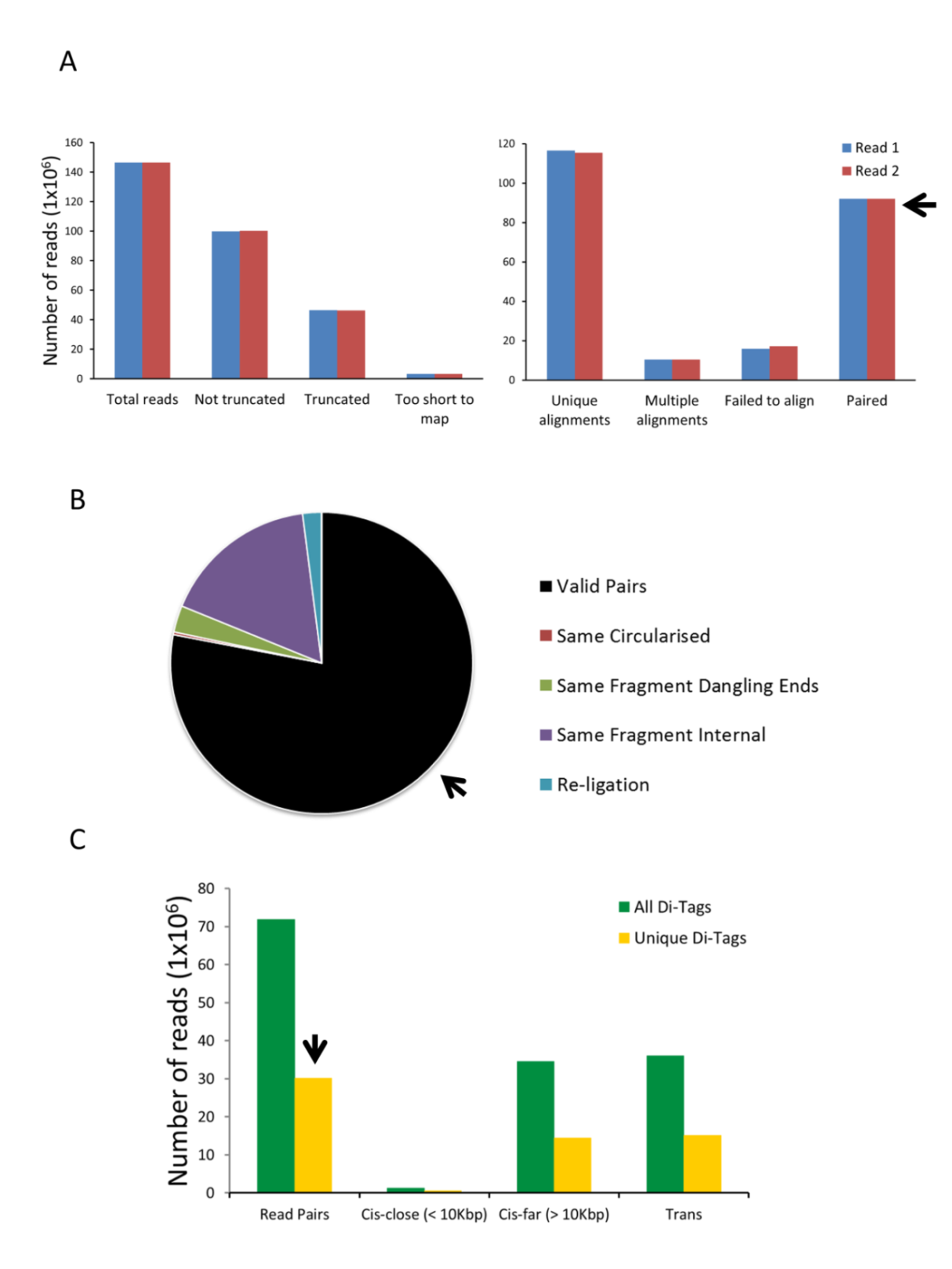  Figure S9 QC report generated after processing of the Myotubes(Day7+AraC) Replicate 2 HiC library.  62.9% (A) of the reads mapped as a valid pair to reference genome, 78% (B) of the mapped reads passed the filtering step. 30 million reads (C) remained after the de-duplication |
| --- |


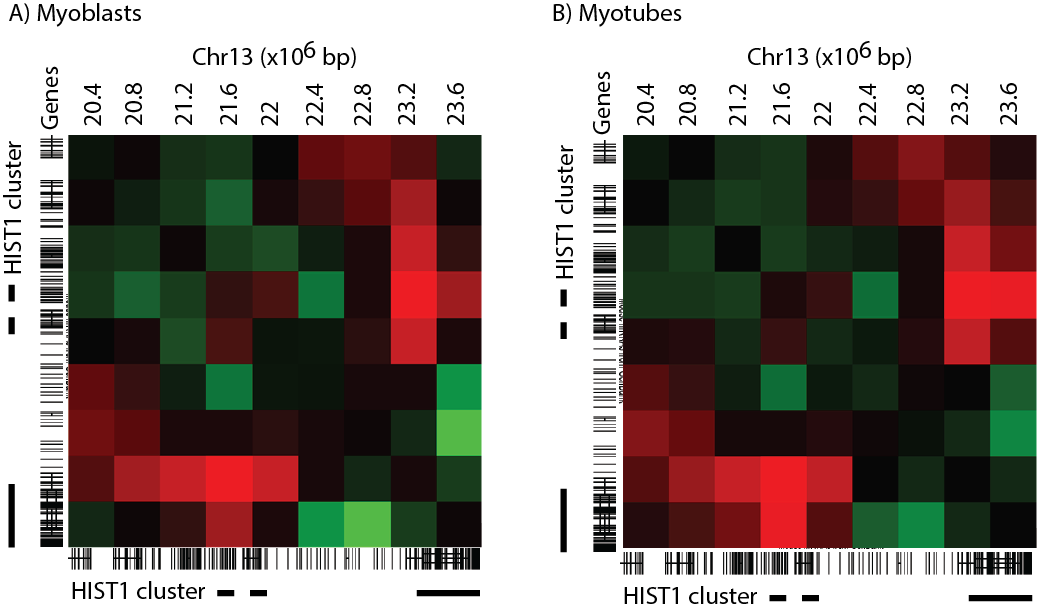


Figure S10 Patches of replication dependent histone variants spatially cluster within the HIST1 locus. Interaction matrices at 400kb resolution. Red indicates high levels of clustering.

| 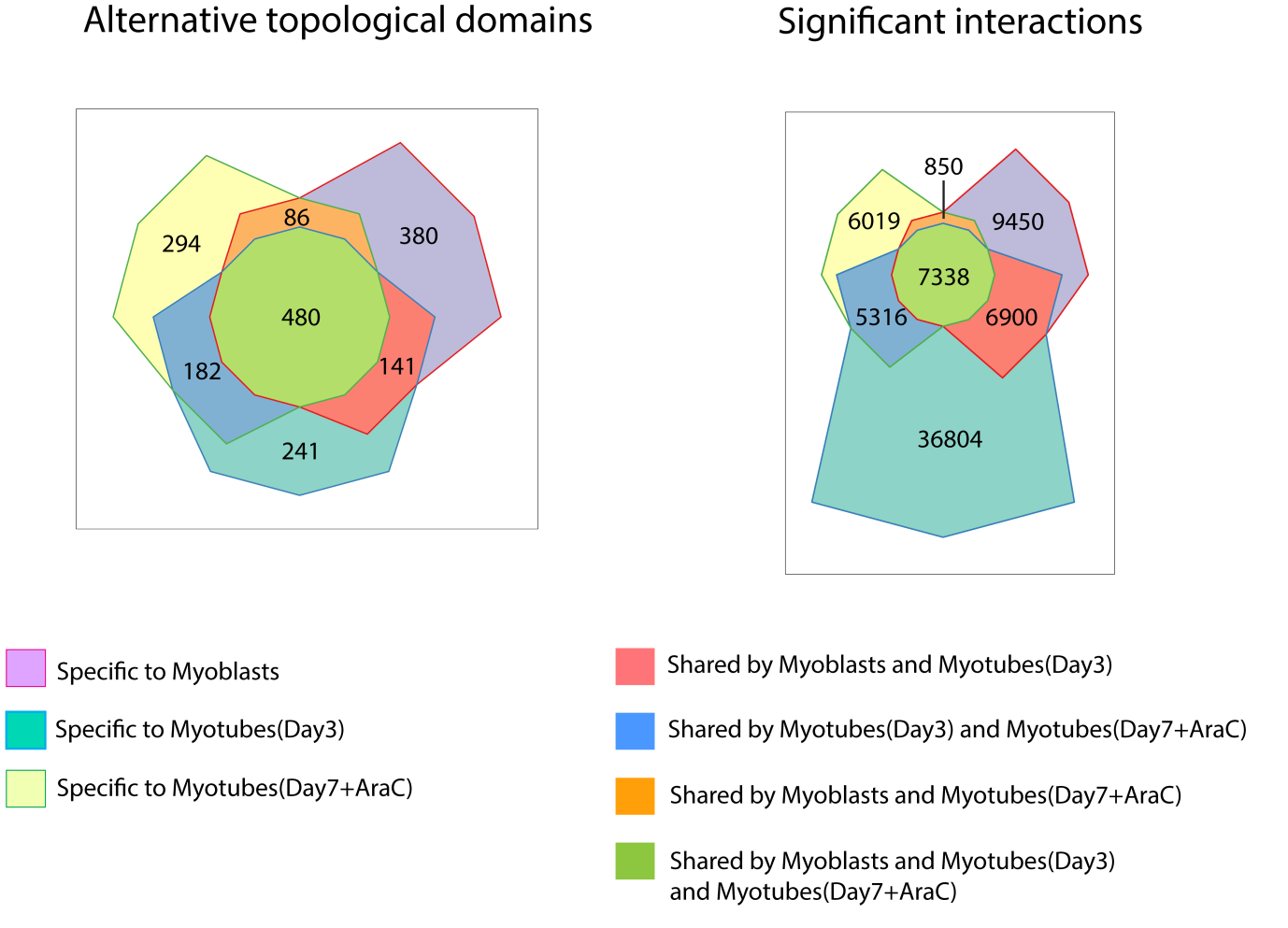  Figure S11 Distributions of shared and unique interactions and TADs across the three conditions.  The number of TADs that were specific or shared between the three conditions was identified using armatus (Filippova et al. 2014) (A). Shared and conserved significant interactions (p value < 0.001, fdr <0.1) were identified using HOMER (B). |
| --- |


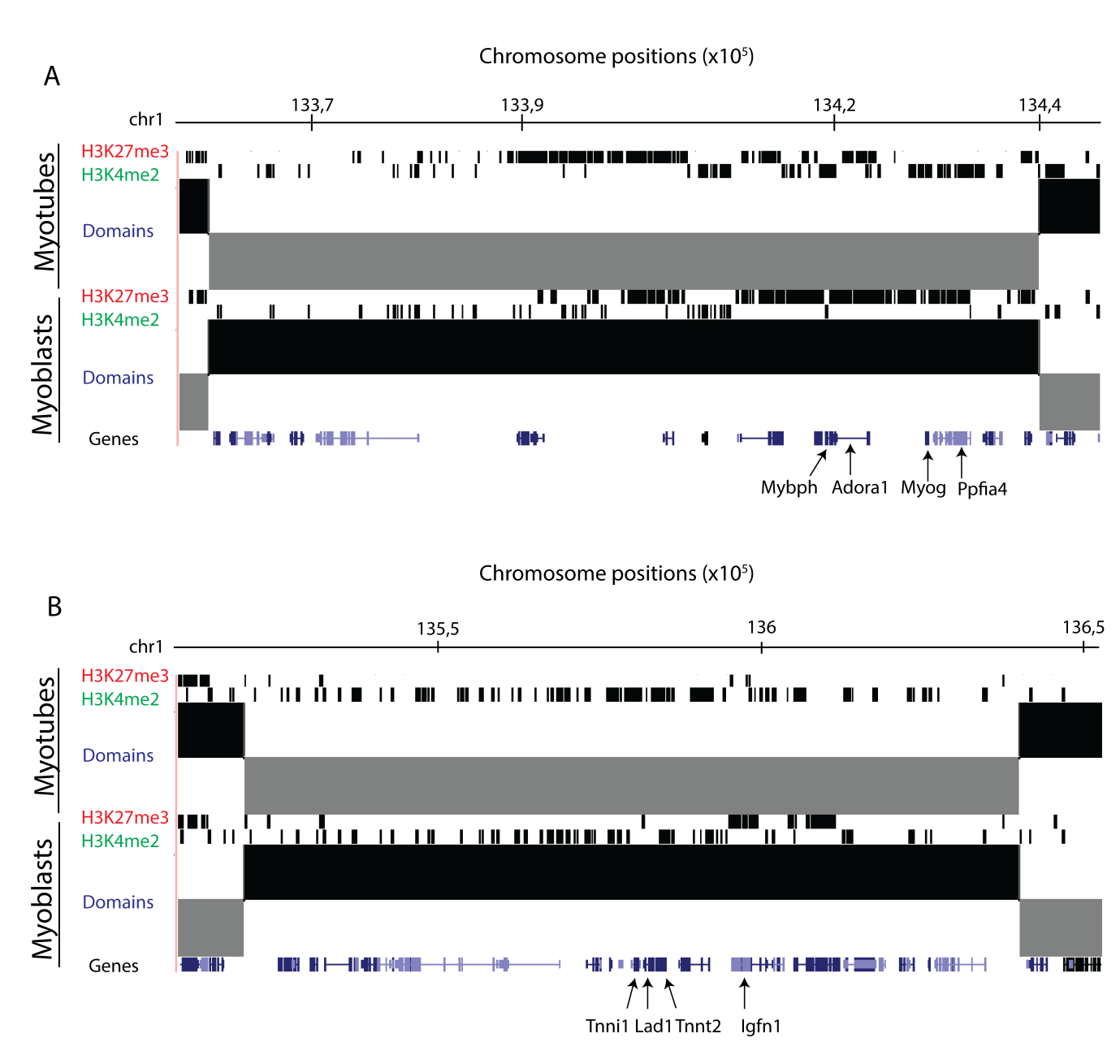


**Figure S12 Two consensus TADs visualized on the UCSC genome browser spanning developmentally regulated arrays of muscle genes and their corresponding histone mark signals on chr1.**
